# A Comparative Study of IVIM‐MRI Fitting Techniques in Glioma Grading: Conventional, Bayesian, and Voxel‐Wise and Spatially‐Aware Deep Learning Approaches

**DOI:** 10.1002/jmri.70301

**Published:** 2026-03-30

**Authors:** Misha P. T. Kaandorp, Andras Jakab, Christian Federau, Peter T. While

**Affiliations:** ^1^ Center for MR Research University Children's Hospital Zurich Zurich Switzerland; ^2^ University of Zurich Zurich Switzerland; ^3^ AI Medical AG Zollikon Switzerland; ^4^ Kyoto Prefectural University of Medicine Kyoto Japan; ^5^ Department of Radiology and Nuclear Medicine St. Olav's University Hospital Trondheim Norway; ^6^ Department of Circulation and Medical Imaging NTNU—Norwegian University of Science and Technology Trondheim Norway

**Keywords:** Bayesian model fitting, deep learning parameter estimation, glioma tumor grading, intravoxel incoherent motion (IVIM), spatially‐aware transformer networks

## Abstract

**Background:**

Intravoxel incoherent motion (IVIM) analysis of diffusion‐weighted MRI (DWI) provides microvascular perfusion and diffusion information. However, parameter estimation is limited by noise sensitivity, variability across fitting methods, and lack of standardization. Deep‐learning (DL)–based approaches, particularly spatially‐aware transformers, may improve robustness, but their clinical utility remains unexplored.

**Purpose:**

To evaluate conventional, Bayesian, and DL–based IVIM fitting methods in glioma patients, focusing on tumor grading accuracy.

**Study Type:**

Retrospective.

**Population:**

Fractal‐noise‐based simulations and preoperative DWI from 20 glioma patients (5 Grade‐2, 3 Grade‐3, 12 Grade‐4).

**Sequence:**

Spin‐echo echo‐planar DWI, 16 *b* values (0–900 s/mm^2^), three orthogonal directions, 3 T.

**Assessment:**

IVIM parameter maps were compared across least squares (LSQ), segmented (SEG), Bayesian shrinkage (BSP), and spatial‐homogeneity (FBM) priors, and DL–based methods, including IVIM‐NET, novel spatially‐aware transformers (NATTEN‐17), and a refined version (SA‐17). Simulation accuracy was evaluated using median absolute percentage error (MDAPE) and bias using median percentage error. In vivo data were visually assessed by the authors for noise suppression and structural preservation. Whole‐tumor diffusion coefficient (*D*), pseudo‐diffusion coefficient (D*), and signal fraction (*f*) values were evaluated across tumor grades and for differentiating Grade‐4 from Grade‐2/3 tumors.

**Statistical Tests:**

Mann–Whitney *U* tests for group comparisons; tumor grading performance using receiver operating characteristic–area under the curve (ROC‐AUC), and pairwise AUC differences using the DeLong test. Significance: *p* < 0.05.

**Results:**

Transformer‐based methods achieved superior simulation accuracy, with significantly lower MDAPE for *f* and D* than all other approaches: NATTEN‐17 (5.91%, 13.31%), SA‐17 (7.73%, 13.66%), LSQ (21.95%, 54.34%), SEG (17.10%, 21.27%), BSP (12.35%, 22.79%), FBM (16.32%, 20.67%). In vivo, they provided superior visual quality and tumor delineation in *f* and D* maps, producing seemingly denoised versions of LSQ, while preserving tumor heterogeneity. The spatially‐aware transformers yielded consistently the highest ROC‐AUC values, particularly for D* (SA‐17: 0.78), significantly outperforming LSQ (0.62), SEG (0.58), FBM (0.62), and IVIM‐NET (0.71).

**Data Conclusion:**

Transformer‐based model fitting has the potential to provide clinically valuable IVIM parameter estimates and improved tumor grading accuracy.

**Evidence Level:**

3.

**Technical Efficacy:**

Stage 2.

## Plain Language Summary

Diffusion‐weighted MRI measures the microscopic motion of water molecules in tissue (diffusion) and can provide information about tumor microcirculation (perfusion). The intravoxel incoherent motion model separates tissue diffusion from microvascular perfusion‐related effects, but its parameter estimates are highly sensitive to image noise and the choice of model fitting method. In this study, we compared conventional fitting methods, Bayesian approaches, and deep learning, specifically novel spatially‐aware transformers that incorporate spatial information. Using computer simulations and preoperative scans from 20 glioma patients, spatially‐aware transformers demonstrated higher accuracy, superior image quality and tumor delineation, and improved tumor grade discrimination.

## 1 Introduction

Diffusion‐weighted MRI (DWI) is a noninvasive imaging technique that primarily detects the random motion of water molecules, offering insights into tissue microstructure and pathology, especially in oncology [1, 2]. Beyond its sensitivity to diffusion, DWI, acquired at weak diffusion sensitization (low *b* values), can also reflect microvascular perfusion effects, an idea originally introduced by Le Bihan et al. [3]. They proposed that water moving through disordered networks of tiny blood vessels produces a signal attenuation similar to diffusion, albeit with a much higher apparent diffusion rate, referred to as the pseudo‐diffusion coefficient (D*). This effect was characterized by the intravoxel incoherent motion (IVIM) model, which aims to quantify both diffusion (*D*) and pseudo‐diffusion components:

(1) $$S\left(b\right)=S_{0}\left(fe^{-bD^{*}}+\left(1-f\right)e^{-\mathrm{bD}}\right),$$

where *f* is the pseudo‐diffusion signal fraction (often referred to as the “perfusion fraction”), *S* is the signal at *b* and *S*_0_ is the signal at *b* = 0. However, despite its promise, IVIM has seen limited clinical implementation due to its dependence on the configuration of MRI acquisition sequences (e.g., number and distribution of *b* values, diffusion‐encoding scheme), sensitivity to noise, lack of standardized protocols, and variabile results depending on the fitting algorithm implemented [4, 5, 6, 7, 8, 9].

Conventionally, parameter estimation for IVIM imaging relies on nonlinear least squares (LSQ) fitting methods [10]. While widely used, LSQ approaches are highly susceptible to noise [10], which has prompted a search for alternative strategies. One such strategy involves breaking the fitting process into stages and estimating diffusion and perfusion components separately, a technique often referred to as segmented fitting [11, 12]. Other efforts have focused on incorporating prior assumptions about spatial consistency, either locally or across the entire image, using probabilistic frameworks such as Bayesian inference [8, 13, 14, 15]. These methods tend to produce cleaner parametric maps than conventional LSQ fitting. More recently, deep learning (DL) techniques have been used for IVIM model fitting [16, 17, 18, 19, 20, 21, 22, 23, 24]. DL methods allow extensive customization in terms of neural network architecture, hyperparameter settings and optimization, training strategy, and training data selection, enabling the development of highly adaptable networks that have the potential to outperform conventional approaches.

When applying IVIM imaging in clinical practice, several limitations of voxel‐wise DL fitting methods should be considered. Training directly on noisy measured data (self‐supervised learning) on a voxel‐by‐voxel basis can yield results that closely mimic conventional LSQ fitting [16, 17, 25]. Furthermore, self‐supervised learning has been shown not to benefit from spatial context [17]. Conversely, training voxel‐wise on simulated ground truth parameter values (supervised learning) can bias estimates toward the mean of the training distribution, particularly under low signal‐to‐noise (SNR) conditions, which may obscure important tissue heterogeneity [16, 25]. This effect is especially pronounced for the estimation of D*, which is known to be ill‐conditioned in tissues with low *f*, such as white matter. As a result, D* estimates are highly sensitive to noise. Since ground truth IVIM parameters are unavailable clinically, supervised DL methods rely on synthetic datasets with known parameters and controlled noise characteristics.

Recently, transformer‐based architectures have been explored for IVIM parameter estimation using supervised learning on synthetic patch‐based datasets, rather than voxel‐wise inputs, with the explicit aim of incorporating spatial context into the fitting process [17]. The underlying motivation is that neighboring voxels often exhibit correlated signal behavior, and access to this spatial information can help mitigate voxel‐wise parameter degeneracy and noise sensitivity inherent to IVIM fitting, particularly for perfusion‐related parameters and in low‐SNR regimes. These transformer‐based networks were shown to reduce supervised training bias, preserve anatomical edges, and improve estimation accuracy and reproducibility across repeated scans, outperforming both voxel‐wise DL and convolution neural network (CNN)‐based approaches while also being more efficient to train for comparable receptive fields [17]. These benefits come with trade‐offs, including the need for carefully designed synthetic training data to avoid bias, longer training times than simpler voxel‐wise networks, and increased computational or memory demands during inference.

Despite these methodological improvements, it remains uncertain whether DL‐based fitting methods offer clinical advantages over conventional techniques. While improved visual quality, structural preservation, and reliable fitting may aid in the assessment of tumor heterogeneity, further validation is needed to determine if DL‐based estimates better reflect tissue biology or improve clinical decision‐making. Most prior studies focus on accuracy, precision, or repeatability in isolation, often comparing only a limited set of estimators without addressing clinically relevant questions such as treatment response or disease characterization.

Thus the aim of this study was to evaluate the performance of several IVIM fitting techniques, including conventional, Bayesian, and voxel‐wise and spatially‐aware DL‐based methods, for estimating IVIM parameters in glioma patients, with a focus on tumor grading accuracy. A further aim was to introduce a refined transformer‐based network that may better preserve structural details.

## 2 Materials and Methods

### 2.1 Datasets

#### 2.1.1 Synthetic Data

Synthetic data provided a well‐defined ground truth, enabling quantitative assessment of estimator performance under ideal conditions containing only diffusion and pseudo‐diffusion signal contributions. Ground‐truth IVIM parameter maps were synthesized using fractal‐noise generation based on Perlin noise [26], as described by Kaandorp et al. [17]. This approach permitted the synthesis of spatial correlations similar to real data. A detailed description can be found in the Supporting Information.

#### 2.1.2 Patient Cohort

Twenty glioma patients (16 males, 4 females; mean age, 52.3 ± 21.3 years; age range, 2–84 years) previously included in earlier studies [27, 28] were included in the present analysis. These studies were approved by the local ethics committee, and the requirement for patient consent was waived. All patients underwent preoperative MRI between 2011 and 2012. The cohort included mainly glioblastoma, oligoastrocytoma, and astrocytoma. Tumors were graded according to World Health Organization (WHO) criteria [29] (5 Grade‐2, 3 Grade‐3, 12 Grade‐4). Corresponding whole‐tumor regions of interest (ROIs) were delineated by two experienced neuroradiologists [27, 28] on IVIM *b = *0 images excluding cystic or necrotic areas, with the help of conventional T1‐weighted pre‐ and post‐contrast (gadolinium) MR images. ROIs in the contralateral normal‐appearing white matter were also defined for control [27]. Imaging was conducted on 3 T MRI scanners (Trio, Verio, or Skyra; Siemens Healthcare, Erlangen, Germany) using a Stejskal–Tanner diffusion‐weighted spin‐echo echo planar imaging sequence with 16 *b* values (0, 10, 20, 40, 80, 110, 140, 170, 200, 300, 400, 500, 600, 700, 800, 900 s/mm^2^) in three orthogonal directions, and the diffusion‐weighted trace image was computed from these measurements (single acquisition per direction).

Fifteen patients were from the cohort in Federau et al. [27], and were acquired with: repetition time (TR) = 4000 ms, echo time (TE) = 99 ms, field of view (FOV) = 297 × 297 mm^2^, acquisition matrix = 256 × 256, 9–21 slices, and receiver bandwidth = 1086 Hz/pixel. Five patients were from the cohort in Federau and O'Brien [28], and acquired with: TR = 4000 ms, TE = 93 ms, FOV = 270 × 270 mm^2^, acquisition matrix = 225 × 225, 20 slices, and receiver bandwidth = 1106 Hz/pixel. Both datasets used a slice thickness of 4 mm, in‐plane resolution of 1.2 × 1.2 mm^2^, 75% partial Fourier encoding, parallel imaging with an acceleration factor of 2, and fat suppression via a spectrally selective saturation routine. Total acquisition time was 3 min 7 s for both studies.

The images were normalized so that the mean intensity within a homogeneous white matter ROI in each patient's *b* = 0 image matched the *S*_0_ values used in the simulations for the corresponding SNR. In this case, the white matter had a mean SNR of approximately 30, corresponding to *S*_0_ = 0.15 in the simulations, with *S*_0_ = 1 corresponding to an SNR of 200. This scaling ensured that network inputs remained within an appropriate range, maintaining approximate equivalence between training and test datasets.

### 2.2 Fitting Methods

The fitting methods described below were applied to both the synthetic and in vivo data, and compared. The conventional and DL methods were implemented in Python and Pytorch, and the Bayesian frameworks in Matlab (R2023b, MathWorks Inc., Natick, MA, USA).

#### 2.2.1 Conventional Estimators

##### 2.2.1.1 Full Nonlinear Least Squares (LSQ)

In this conventional approach, all IVIM parameters are jointly optimized using a nonlinear least‐squares algorithm, specifically employing the trust‐region‐reflective solver [10]. This algorithm allows for the enforcement of predefined parameter bounds. The upper bound of *f* was 100%, whereas the upper bounds of *S*_0_, *D*, and D* were unconstrained. D* was set to > 3 × 10^−3^ mm^2^/s to avoid nonphysiological output, while all other parameters (*S*_0_, *D*, *f*) were constrained by a lower bound of 0. Note that essentially identical results were obtained from LSQ if instead the bounds of LSQ were set equal to the tighter limits of the uniform distributions used for training the networks (see section on NATTEN‐17 below).

##### 2.2.1.2 Segmented Approach (SEG)

In the segmented approach [11, 12], it is assumed that the influence of pseudo‐diffusion becomes negligible beyond a certain *b*‐value threshold, typically set at 200 s/mm^2^. Under this assumption, the signal decay at high *b* values can be modeled as a mono‐exponential function, allowing for a direct estimation of *D*. The signal extrapolated to *b* = 0 from this fit, often referred to as S_int_, is then used to approximate *f*. Finally, D* is estimated by fitting the full IVIM model again using LSQ, either fixing both *D* and *f* or only *D*. In this study, only *D* was fixed, and both *f* and D* were estimated in the second step.

#### 2.2.2 Bayesian Frameworks

##### 2.2.2.1 Bayesian Gaussian “Shrinkage” Prior (BSP)

Bayesian estimation combines prior knowledge or assumptions with observed data to yield posterior parameter distributions, from which summary statistics (e.g., mean, mode) provide the parameter estimates [10]. A Gaussian prior is commonly used, but assigning fixed distributions is challenging in clinical IVIM, where reliable a priori information may be lacking. Instead, Orton et al. [13] proposed a *shrinkage prior*, where the mean and variance of the Gaussian prior are estimated from voxel groups (e.g., within a lesion) and refined iteratively during Markov Chain Monte Carlo (MCMC) sampling. This approach reduces outlier effects by “shrinking” noisy voxel estimates toward the group mean, thereby improving stability under parameter degeneracy. The following BSP variants were applied: (i) *BSP*_*image*_ where the prior is based on the entire image (used for the synthetic data) (ii) *BSP*_*brain*_, where the prior is based on all voxels in the brain (in vivo), (iii) *BSP*_*tumor*_, where the prior is based on the whole 3D tumor ROIs (i.e., all slices). Each MCMC run included 2000 burn‐in and 20,000 sampling steps. Further information can be found in While [8].

##### 2.2.2.2 Spatial Homogeneity Prior—Fusion Bootstrap Moves (FBM)

The FBM approach [14] regularizes voxel estimates by incorporating a neighborhood‐based regularization term into the parameter estimation, controlled by a tuning parameter *α*. Low *α* yields maps closer to the chosen conventional fitting procedure (independent voxels), whereas high *α* enforces localized smoothing. To balance across parameters with different scales, a weighting vector was applied containing the inverses of the following parameter values: *D* = 1 × 10^−3^ (mm^2^/s); *f* = 0.1; D* = 10 × 10^−3^ (mm^2^/s). For the datasets in this study, *α* = 1. Initial estimates were seeded by either LSQ (*FBM*_*LSQ*_) or SEG (*FBM*_*SEG*_), followed by bootstrap resampling of voxel signals. Candidate updates derived from this bootstrapping were integrated via “fusion moves,” minimizing an energy function to refine the parameter maps. Further information can be found in While [8]. Results for FBM_LSQ_ are included in the Supporting Information.

#### 2.2.3 Deep Learning

##### 2.2.3.1 IVIM‐NET_optim_

IVIM‐NET_optim_ [18] is a self‐supervised DL architecture, which was proposed as a refinement to IVIM‐NET_orig_ [19] (see Supporting Information for details). IVIM‐NET_optim_ estimates *S*_0_, *D*, *f*, and D*, and uses parallel multi‐layer perceptron (MLP) sub‐networks for *S*_0_, *D*, *f*, and D*, each with two hidden layers (units = number of *b* values), batch normalization, 10% dropout, exponential linear unit (ELU) [30] activations, and sigmoid outputs to enforce physiological constraints. The network is trained in a self‐supervised manner directly on measured DWI signals, using a physics‐informed loss between this measured signal and the signal predicted from the network's parameter estimates using Equation (1). Training used 500 batches per epoch, with batch size 128, and Adam [31] optimization at a learning rate of 3 × 10^−5^. Early stopping was enforced after 10 non‐improving validation epochs, which typically occurred around 100 epochs.

##### 2.2.3.2 NATTEN‐17

NATTEN‐17 [17] involves spatially‐aware transformer architectures. NATTEN‐17 consists of eight neighborhood attention (NA) [32] blocks with 128 hidden units in each attention block. Each block has a kernel size of 3, such that the convolution‐like nature of the set of eight blocks results in a total receptive field of 17 × 17. NATTEN‐17 is trained supervised on patches of DWI signals (17 × 17 × #*b*‐values), which were derived from simulated IVIM parameters.

To generate the synthetic training data, for each patch, the center pixel was assigned a randomly sampled combination of IVIM parameters drawn from uniform distributions with the following bounds: 0 ≤ *S*_0_ ≤ 1, 0 × 10^−3^ ≤ *D* ≤ 3 × 10^−3^ mm^2^/s, 0 ≤ *f* ≤ 50%, and 3 × 10^−3^ ≤ D* ≤ 100 × 10^−3^ mm^2^/s. A random subset of the other 288 pixels in the patch was assigned identical parameters to the center pixel (termed “correlated neighbors”), while the remaining voxels were assigned a second independent set of parameters drawn from the same uniform distributions. This set of synthetic training data is referred to as “random‐uniform”. The patch propagation and attention mechanisms of NATTEN‐17 are illustrated in the Supporting Information, Figure S1A.

The synthetic DWI data patches used for training were generated at the same 16 *b* values used in the in vivo acquisition scheme. Rician noise was added with the noise level set such that *S*_0_ = 1 corresponded to an SNR of 200. Training samples were generated on‐the‐fly, enabling the presentation of an effectively unlimited variety of unique patches. The network was trained for 300 epochs of 25,000 batches each (equivalent to 15,000 epochs under the 500‐batch convention), using the Adam optimizer with a learning rate of 1 × 10^−4^.

##### 2.2.3.3 SA‐17

In this study, SA‐17, a modified version of NATTEN‐17, is introduced in which the eight NA blocks are replaced by three self‐attention (SA) [33] blocks. While NATTEN‐17 uses a convolution‐like approach restricted to a fixed local neighborhood of 3 × 3 in each NA block, SA blocks attend to all voxels within the input patch in each block. The hypothesis here is that SA‐17 can capture long‐range spatial dependencies more efficiently as it has access to all neighbors in each SA block. By operating on individual batches of patches of the image, SA‐17 can be applied on images without exceeding memory limits, albeit at the cost of requiring patch extraction at inference. SA‐17 was trained supervised using the same synthetic training dataset (i.e., random‐uniform patches) and optimization protocol of the DWI data as NATTEN‐17. The patch propagation and attention mechanisms of SA‐17 are illustrated in the Supporting Information, Figure S1B.

##### 2.2.3.4 SA‐1

For comparison, a supervised SA‐1 network was also trained, an identical network to SA‐17 (i.e., 3 SA blocks), but applied to 1 × 1 random‐uniform patches (i.e., single pixels), effectively reducing the architecture to a voxel‐wise network (i.e., with no access to spatial information), but with attention blocks.

In the Supporting Information, we additionally report methodology and results for SA‐1 and SA‐17 trained on random patch‐based datasets generated using IVIM parameter values sampled from Gaussian distributions rather than uniform distributions. These Gaussian distributions are guided by literature values and empirical observations, as described in Kaandorp et al. [17], and are referred to here as “random‐gaussian” training. By comparing networks trained on random‐uniform and random‐gaussian patches, we assess whether increasing the receptive field of the supervised self‐attention network reduces bias toward the mean of the training distribution and improves robustness to variations in prior parameter assumptions.

### 2.3 Evaluation

#### 2.3.1 Evaluation in Synthetic Data

Performance of the estimators on the synthetic data was evaluated using a combination of visual inspection by the authors and quantitative metrics. Specifically, the resulting IVIM parameter maps (*D*, *f*, D*) as well as root‐mean‐square error (RMSE) and absolute error maps were visually compared by the authors to assess spatial fidelity, noise characteristics, and the presence of bias across methods. The RMSE was computed (for each voxel) between the predicted DWI signals derived from the estimated parameters (using Equation 1) and the corresponding ground truth signals.

For quantitative evaluation, median absolute percentage error (MDAPE) and median percentage error (MDPE) were employed, defined as:

(2) $$\text{MDAPE}\left(\theta_{1:N}\right)=\text{Median}\left(\left|\frac{\theta_{i}-\theta_{T,i}}{\theta_{T,i}}\right|\right)\times 100,$$

(3) $$\text{MDPE}\left(\theta_{1:N}\right)=\text{Median}\left(\frac{\theta_{i}-\theta_{T,i}}{\theta_{T,i}}\right)\times 100,$$

where $\theta_{i}$ denotes one of the three IVIM parameters (*D*, *f*, D*) at each pixel *i*, and $\theta_{T,i}$ the corresponding ground truth parameter estimate. MDAPE is a measure of accuracy, whereas MDPE is a measure of bias. Statistical significance of differences in errors between methods was tested using a Mann–Whitney *U* test with Bonferroni correction. A *p* value < 0.05 was considered significant.

#### 2.3.2 Evaluation in Tumor Cohort

Clinical performance was evaluated in the tumor cohort using a combination of descriptive visual inspection and quantitative analysis. Visual examination of IVIM parameter maps (*D*, *f*, D*) and corresponding RMSE maps was performed by the authors and presented for representative cases from one Grade‐2 and one Grade‐4 tumor patient. Specifically, the following features were examined across estimation methods: (1) the visual differentiation between tumor tissue and surrounding parenchyma, particularly in *f* and D* maps; (2) spatial smoothness and noise characteristics of the parameter maps; (3) degree of structural detail and tumor heterogeneity; and (4) presence of apparent systematic bias or artifacts, such as globally elevated or suppressed parameter values.

We further quantitatively analyzed estimated parameters within whole tumor ROIs across tumor grades, as described below (Statistical Analysis). For the tumor cohort, the time required for the conventional estimators to be performed, as well as the training and inference time for the network‐based methods, was recorded.

### 2.4 Statistical Analysis

A Mann–Whitney *U* test with Bonferroni correction was used to test for significant differences between tumor grades. A *p* value < 0.05 was considered significant. The differentiation ability of IVIM parameters was assessed using receiver operating characteristic (ROC) curves and the area under the curve (AUC) to distinguish Grade‐4 from combined Grade‐2 and Grade‐3 tumors, based on all voxels within the tumor ROIs. DeLong testing [34] was performed to compare the AUC values for significance between the different fitting methods. Additionally, we performed a joint analysis combining diagnostic accuracy in the tumor cohort with estimation bias observed in the synthetic data.

As tumor heterogeneity may impact grading, we further explored grading performance using percentile‐based subregion analysis. Specifically, for each IVIM parameter (*D*, *f*, D*), we computed AUC values not only for the whole tumor ROIs but also for voxels corresponding to selected percentile values (5%, 25%, 50%, 75%, 95%) of the parameter distribution within the tumor. This approach evaluates whether grading accuracy can be improved by focusing on subregions with particularly low or high parameter values.

## 3 Results

The following results are for LSQ, SEG, BSP, FBM_SEG_, IVIM‐NET_optim_, NATTEN‐17, SA‐17, SA‐1. Results for FBM_LSQ_ and IVIM‐NET_orig_, as well as for SA‐17 and SA‐1 trained on the “random‐gaussian” synthetic data, can be found in the Supporting Information.

### 3.1 Evaluation in Synthetic Data

**FIGURE 1:**
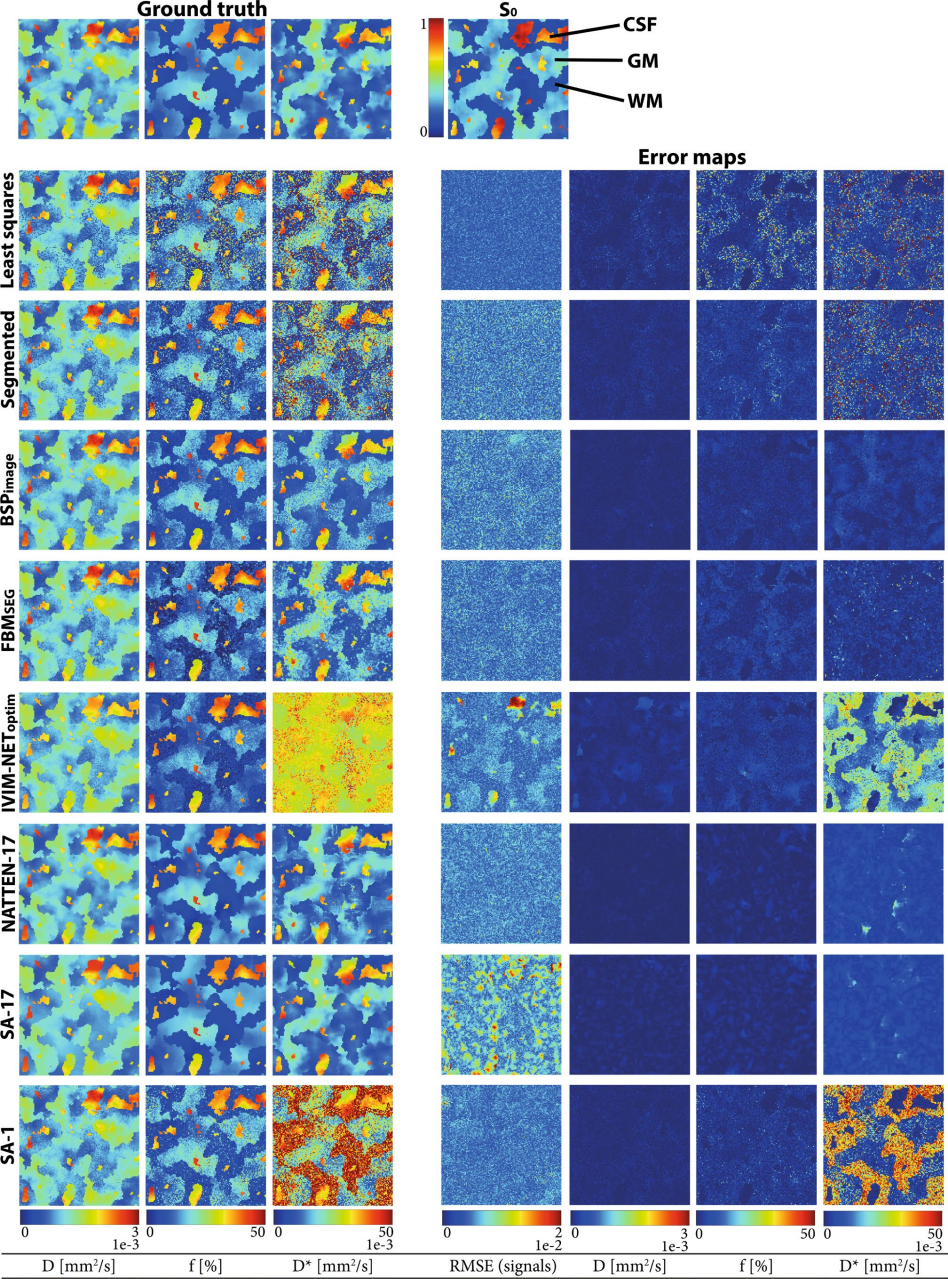
Example fractal‐noise based IVIM parameter maps, root mean square error (RMSE) maps (calculated between the estimated DWI signal and the ground truth DWI signal), and absolute error maps, estimated for eight of the evaluated IVIM fitting methods. Corresponding maps of the ground truth (*left top*; CSF, cerebral spinal fluid; GM, gray matter; WM, white matter) are also shown. (D*, pseudo‐diffusion coefficient; *D*, diffusion coefficient; *f*, perfusion fraction).

**FIGURE 2:**
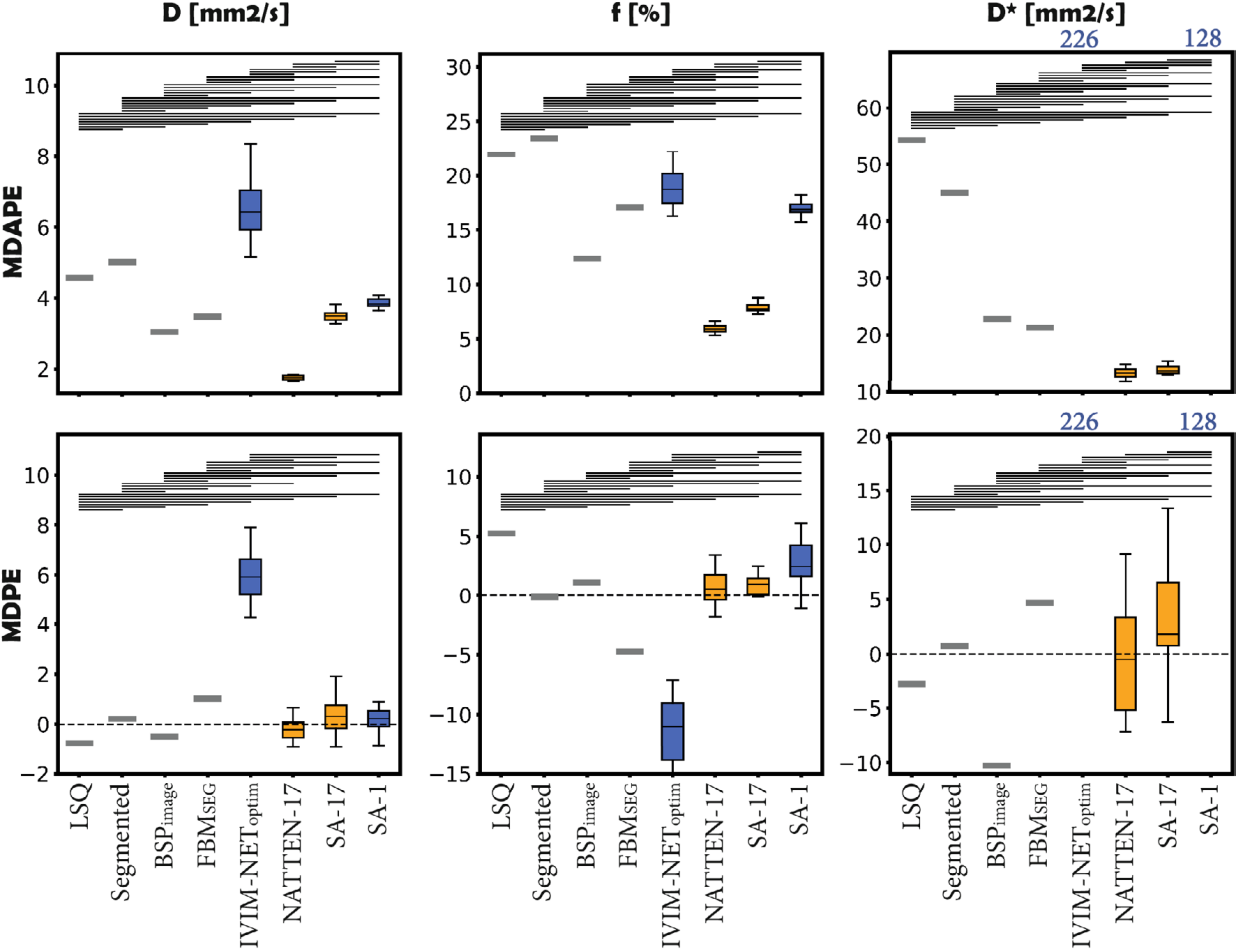
Boxplots showing the median absolute percentage error (MDAPE) and median percentage error (MDPE) for each IVIM parameter for eight of the evaluated IVIM fitting methods, calculated on the entire IVIM fractal‐based test set (40 sets of fractal‐noise IVIM parameter maps). To provide insight into network stability, the error metrics were computed for the spatially‐aware transformer networks at each epoch of the last 20 epochs of the entire training. For IVIM‐NET, these were run 20 times. Gray represents conventional and Bayesian estimators, blue represents voxel‐wise DL‐based estimators, orange represents spatially‐aware DL estimators. A Mann–Whitney *U* test with Bonferroni correction was used to test for significant differences between groups (indicated by bars above the plots). Values printed above the boxplots indicate median values that fell outside the displayed plot range. (D*, pseudo‐diffusion coefficient; *D*, diffusion coefficient; *f*, perfusion fraction).

Figures 1 and 2 summarize the performance of eight of the estimators on fractal‐noise‐based IVIM parameter maps. Figure 1 shows parameter maps, RMSE, and absolute error maps for a representative instance of fractal‐noise‐based synthetic data. Figure 2 provides quantitative evaluation metrics (MDAPE and MDPE), which are consistent with the visual observations from Figure 1. Across all comparisons, the spatially‐aware transformer networks (NATTEN‐17, SA‐17) achieved the closest match to the ground truth (top panels of Figure 1) and consistently yielded significantly lower errors. Within this group, NATTEN‐17 attained significantly lower error values than SA‐17, although both produced visually similar parameter maps. However, on visual inspection, we contend that SA‐17 preserved boundary structures better, even though its RMSE values were higher.

LSQ and SEG showed generally inferior performance, producing visually noisier maps, particularly for D*. SA‐1 exhibited pronounced bias for D*. IVIM‐NET_orig_ was unstable and performed poorly across parameters (Supporting Information, Figures S1 and S2), whereas IVIM‐NET_optim_ showed improved stability but demonstrated high biases for D*, similar to SA‐1. Bayesian approaches generally outperformed LSQ and SEG. The FBM method generated smoother parameter maps, with the FBM_seg_ variant yielding the most visually coherent maps and significantly the lowest errors, particularly for D* (see Supporting Information for FBM_LSQ_). BSP produced the cleanest parameter maps and significantly the lowest errors among the Bayesian methods, although its overall error remained significantly higher than that of the spatially‐aware transformer networks.

### 3.2 Tumor Cohort Analysis

**FIGURE 3:**
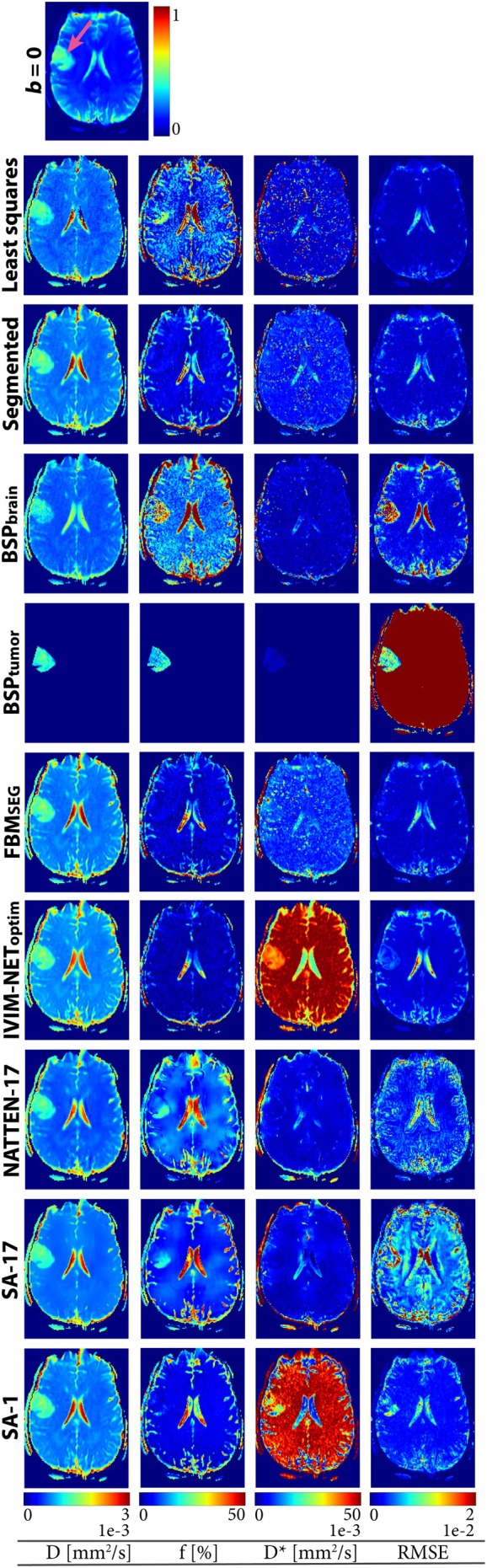
IVIM parameter maps and root mean square error (RMSE) maps (calculated between the estimated DWI signal and the ground truth DWI signal) for a representative slice of a Grade‐2 oligoastrocytoma patient. The maps are estimated for nine of the evaluated IVIM fitting methods. Also shown is the *b* = 0 image (*top left*), where the purple arrow indicates the location of the oligoastrocytoma. Note the heterogeneous high *f* in the tumor region of the spatially‐aware networks, also observed for LSQ (with noise) and BSP variants, but not for the other approaches. (D*, pseudo‐diffusion coefficient; *D*, diffusion coefficient; *f*, perfusion fraction).

Figure 3 shows a representative slice from a patient with a Grade‐2 tumor. Across the estimators, *D* maps were generally similar, while *f* maps fell into two broad categories: those that on visual inspection clearly delineated the tumor (including LSQ, BSP_brain_ BSP_tumor_, NATTEN‐17, and SA‐17) and those that did not (including SEG, FBM_SEG_, IVIM‐NET_optim_, and SA‐1). D* maps were also largely similar across methods, with the notable exception of SA‐1 and IVIM‐NET_optim_, which showed pronounced bias.

**FIGURE 4:**
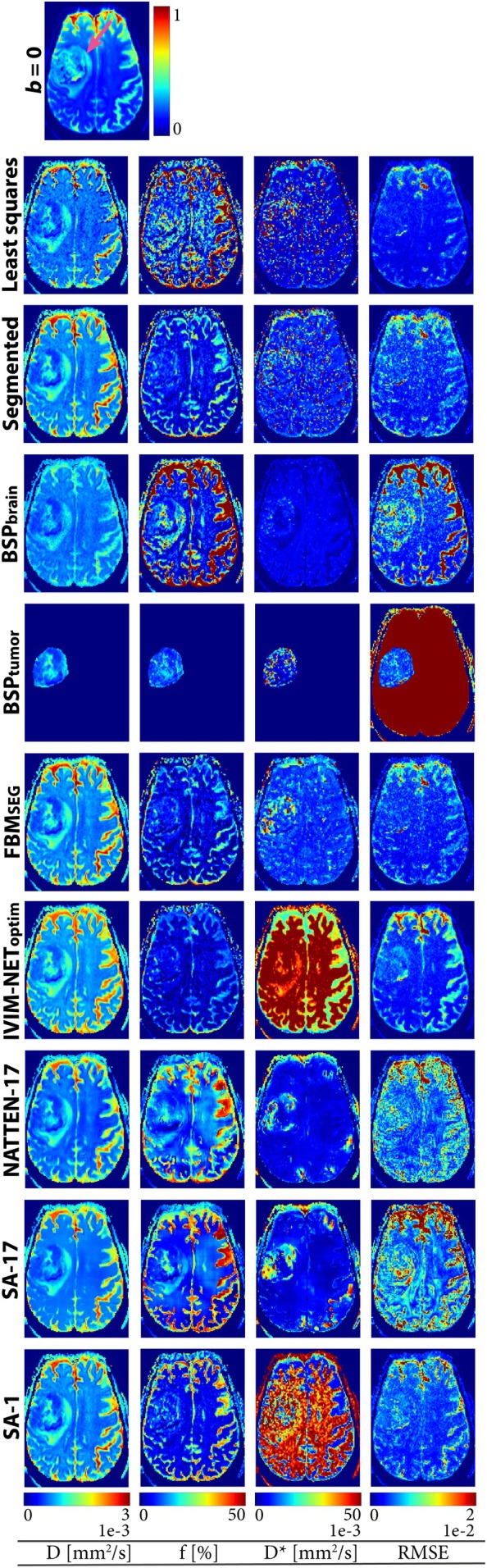
IVIM parameter maps and root mean square error (RMSE) maps (calculated between the estimated DWI signal and the ground truth DWI signal) for a representative slice of a Grade‐4 glioblastoma patient. The maps are estimated for nine of the evaluated IVIM fitting methods. Also shown is the *b* = 0 image (*top left*), where the purple arrow indicates the location of the glioblastoma. Note the strong edge preservation for the SA‐17 network, particularly for *f*, and the high D* estimated in the tumor for SA‐17 and NATTEN‐17. (D*, pseudo‐diffusion coefficient; *D*, diffusion coefficient; *f*, perfusion fraction).

Figure 4 presents results from a Grade‐4 tumor. Overall, the previous observations largely hold, with *D* and *f* maps showing similar patterns as in Figure 3. Here, SA‐17 shows smooth but detailed and heterogeneous *f* in the tumor region. The main differences arise in the D* maps: several estimators, including the spatially‐aware networks and BSP methods, now also highlighted tumor regions with elevated D*, in addition to *f*. Among these, we contend on visual inspection that SA‐17 preserved structural detail and tissue heterogeneity better than NATTEN‐17. Bayesian FBM methods continued to provide cleaner maps than LSQ and SEG, while BSP_brain_ and BSP_tumor_ reproduced spatial patterns in *f* closely matching those obtained with the spatially‐aware networks. Overall, the spatially‐aware transformer networks produced smooth yet detailed parameter maps, resembling denoised LSQ outputs.

**FIGURE 5:**
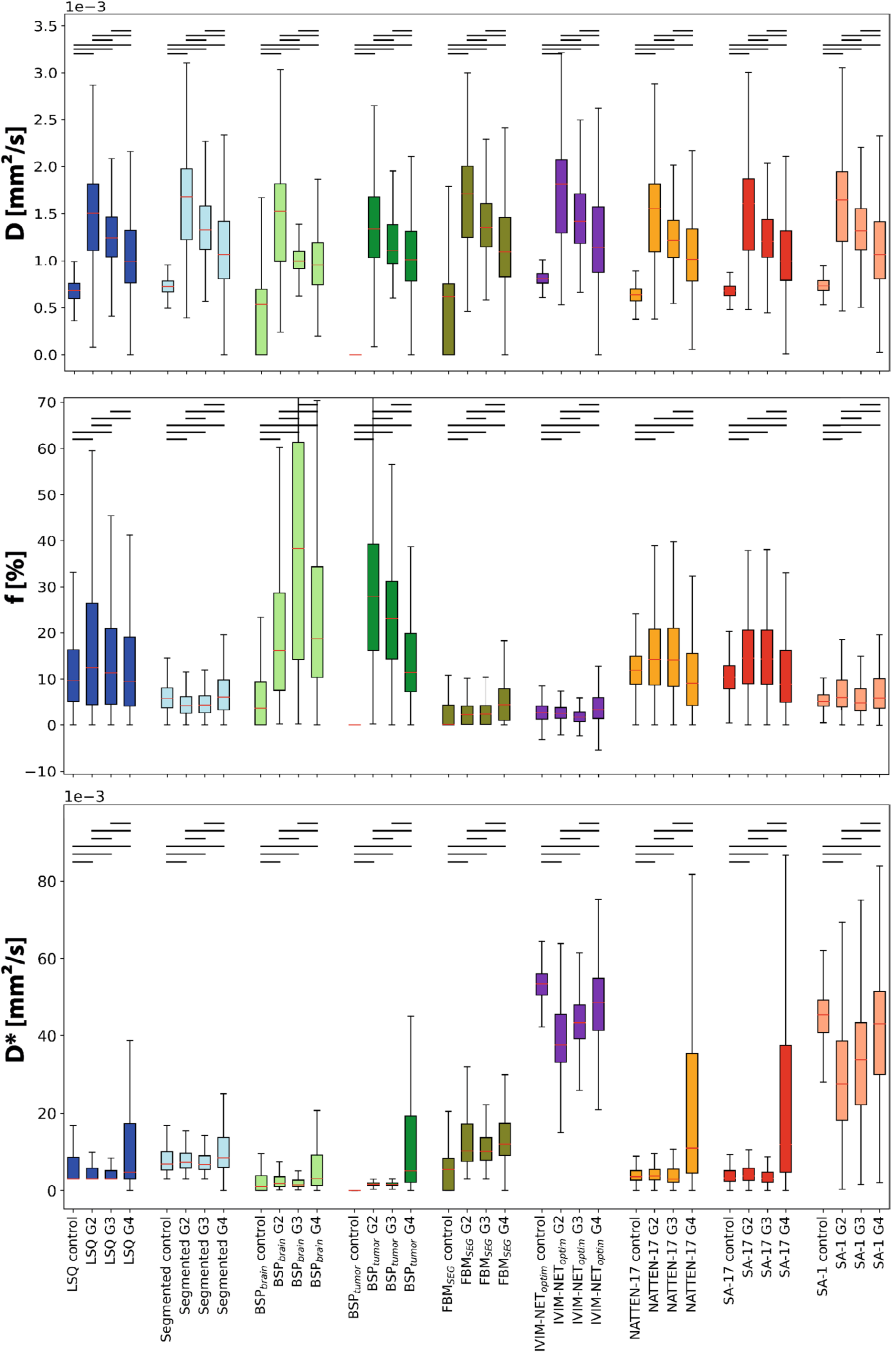
Box‐and‐whisker plots for the IVIM parameters of all pooled estimates within the whole tumor ROIs for nine of the evaluated IVIM fitting methods, across the different tumor grades and contralateral control region. A Mann–Whitney *U* test with Bonferroni correction was used to test for significant differences between groups (indicated by bars above the plots). (D*, pseudo‐diffusion coefficient; *D*, diffusion coefficient; *f*, perfusion fraction).

Figure 5 further illustrates tumor‐grade‐dependent differences across methods, where for each parameter, estimates were pooled from all the voxels within the whole tumor ROIs across tumor grades for all patients. All estimation methods showed a significant reduction in D values with increasing tumor grade (also significantly different from the lower values observed in the control regions). For *f*, the spatially‐aware networks produced a pattern of significantly lower values in Grade‐4 tumors, accompanied by significantly elevated D*. Conversely, SEG produced slightly higher *f* and D* for Grade‐4 tumors. SA‐1 again showed high D* (all grades), mirroring its simulation performance and suggesting systematic bias, and IVIM‐NET_optim_ exhibited similar deviations. Bayesian estimators also displayed distinct grade‐related trends: FBM methods resulted in slightly higher *f* and D* for Grade 4 tumors, while BSP_tumor_ yielded lower *f* and higher D* in this group.

**FIGURE 6:**
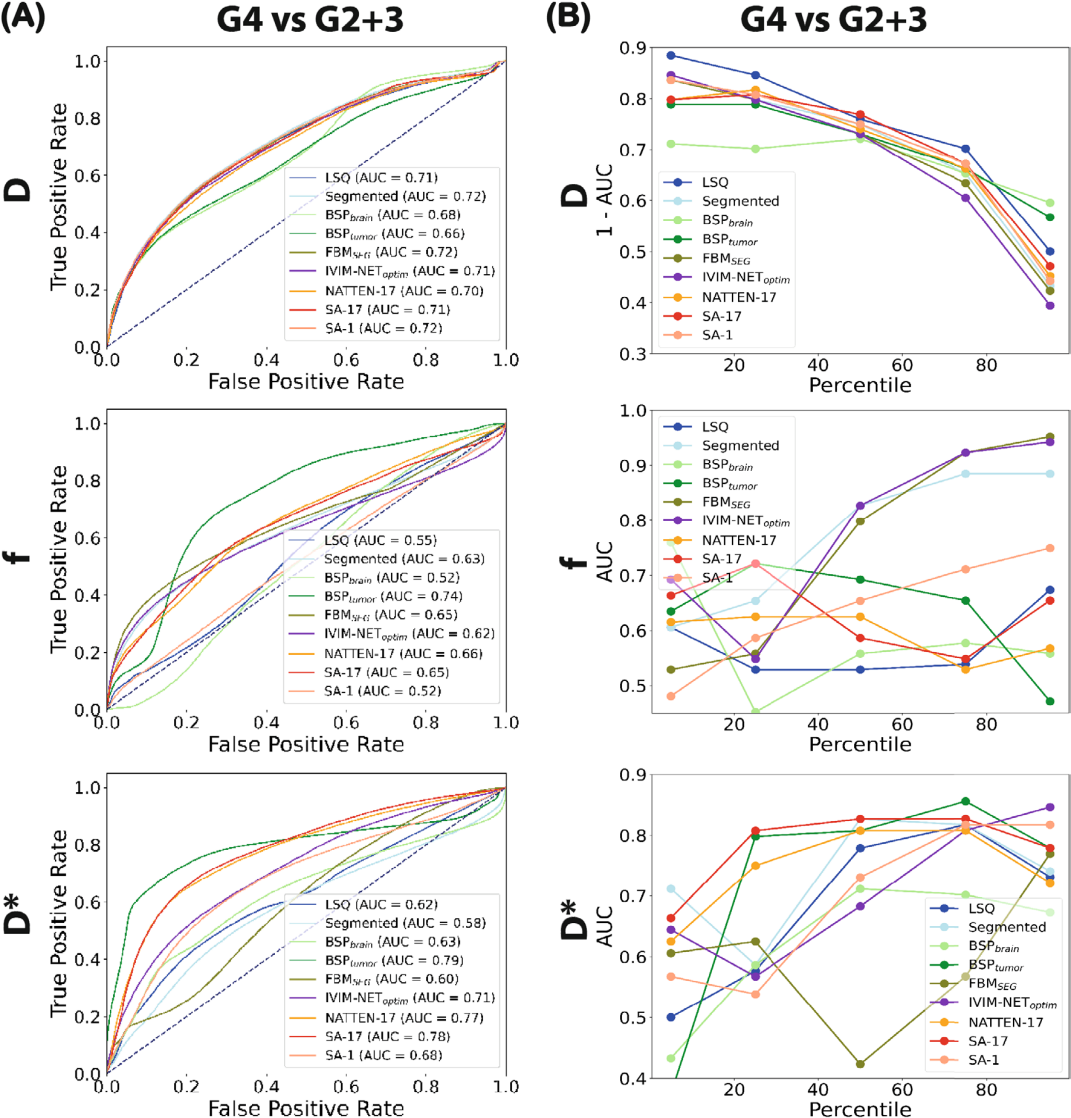
ROC curves and AUC values for differentiating Grade‐4 from combined Grade‐2 and Grade‐3 tumors for nine of the evaluated IVIM fitting methods using (A) parameter estimates from all voxels within the whole tumor ROIs, and (B) percentile values (5%, 25%, 50%, 75%, 95%) from the estimates for each tumor ROI. The AUC values in (B) suggest improved grading accuracy may be possible by focusing on subregions with low *D*, high *f*, and/or high D*. (D*, pseudo‐diffusion coefficient; *D*, diffusion coefficient; *f*, perfusion fraction).

Figure 6A shows ROC curves for distinguishing Grade‐4 from combined Grade‐2 and Grade‐3 tumors, based on all voxels within the tumor ROIs. Using the DeLong test, AUCs for all parameters differed significantly between the methods, with the exception of parameter *f* between FBM_SEG_–NATTEN‐17 and BSP_brain_–SA‐1. Overall, these results support discriminative performance of the spatially‐aware transformer networks, which consistently ranked among the top‐performing estimators across all parameters. For the SA‐17 network trained on a uniform distribution, the results were AUC_D_ = 0.71, AUC_f_ = 0.65, and AUC_D__*_ = 0.78; when trained on a Gaussian distribution (see also Supporting Information, Figure S6), performance improved slightly for the pseudo‐diffusion parameters (AUC_D_ = 0.70, AUC_f_ = 0.67, AUC_D*_ = 0.80). In contrast, LSQ (AUC_D_ = 0.71, AUC_f_ = 0.55, AUC_D*_ = 0.62) and SEG (AUC_D_ = 0.72, AUC_f_ = 0.63, AUC_D__*_ = 0.58) demonstrated significantly lower discriminative ability for the pseudo‐diffusion parameters compared to the transformer‐based methods for all parameters. IVIM‐NET_optim_ (AUC_D_ = 0.71, AUC_f_ = 0.62, AUC_D*_ = 0.71) achieved significantly lower performance than the spatially‐aware networks. Among the Bayesian approaches, FBM methods showed significantly lower discriminative power for D* relative to the spatially‐aware networks; however, FBM_LSQ_ performed significantly better than FBM_SEG_ for this parameter (AUC_D*_: 0.68 vs. 0.60, respectively). BSP_tumor_ exhibited significantly the highest discriminative capability for the pseudo‐diffusion parameters, achieving AUC_f_ = 0.74 and AUC_D*_ = 0.79, yet its AUC_D_ was significantly lower than that of all other approaches (AUC_D_ = 0.66).

Figure 6B shows AUC values calculated for differentiating Grade‐4 from combined Grade‐2 and Grade‐3 tumors for all estimators using percentile values (5%, 25%, 50%, 75%, 95%) from all the estimates for each tumor ROI, suggesting that improved grading accuracy may be possible if analysis is restricted to tumor subregions of low *D*, high *f* and/or high D*. There was strong discriminative power observed for *f* from SEG (0.88), FBM_SEG_ (0.95), and IVIM‐NET_optim_ (0.94) at higher percentiles (95%), whereas the spatially‐aware networks and BSP_tumor_ showed strong discriminative power for D* at all but the lowest percentile (5%).

**FIGURE 7:**
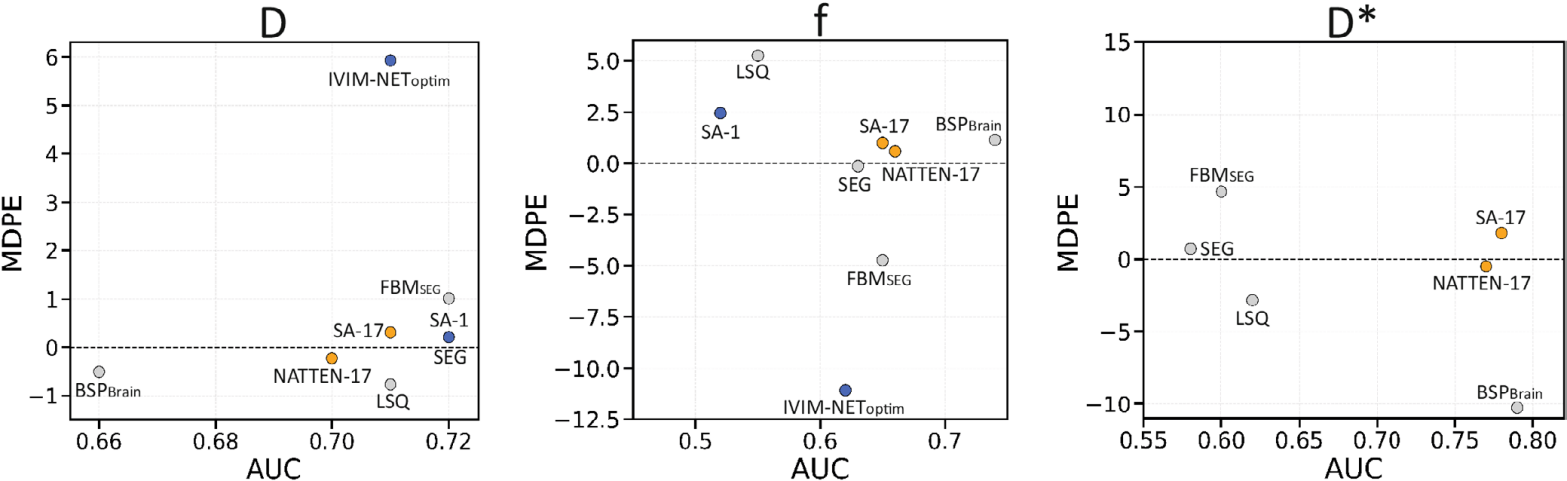
Comparison of diagnostic performance against simulation bias for nine evaluated IVIM fitting methods. For each IVIM parameter, the receiver operating characteristic area under the curve (ROC‐AUC) for differentiating Grade‐4 from combined Grade‐2 and Grade‐3 tumors is plotted against the median percentage error (MDPE) obtained from the fractal‐based simulation test set. Each marker represents one fitting method. Gray represents conventional and Bayesian estimators, blue represents voxel‐wise DL‐based estimators, orange represents spatially‐aware DL estimators. The horizontal dashed line indicates zero bias (MDPE = 0). The D* panel does not include IVIM‐NET_optim_ and SA‐1 because of their extremely high biases (MDPE: IVIM‐NET_optim_ = 226%, SA‐1 = 128%). (D*, pseudo‐diffusion coefficient; *D*, diffusion coefficient; *f*, perfusion fraction).

Figure 7 illustrates the relationship between tumor grading performance (ROC‐AUC) for whole‐tumor ROIs and simulation bias (MDPE) for each fitting method and IVIM parameter. Overall, the spatially‐aware transformer‐based methods consistently occupied the most favorable regions of the joint AUC–bias space, particularly for the perfusion‐related parameters, outperforming the other estimators. BSP_brain_ showed high AUC and low bias for *f*, but high bias in D*. LSQ performed substantially worse for *f* and D*. SEG showed low bias for *f* but lower AUC than several other approaches, and also performed substantially worse for D*.

Table 1 summarizes the computational times of the different estimators. SA‐17 required substantially less training time (21 h) compared to NATTEN‐17 (8 days), and 2 h for inference across all patients, whereas NATTEN‐17 achieved faster inference (20 min). IVIM‐NET_optim_ achieved the shortest times overall, with training completed in 210 s and inference in 80s (average over 20 runs). In contrast, the conventional estimators required considerably longer application times. The Bayesian estimators were the most time‐consuming, and were only applied to those slices containing tumor, that is, 180 out of 392 slices.

**TABLE 1:** Computation times for nine of the evaluated IVIM fitting methods when applied to the cohort of 20 glioma patients.

	Training	Inference/application
LSQ	N/A	10 h
SEG	N/A	9 h
BSP_brain_	N/A	59 h
BSP_tumor_	N/A	3 h
FBM_SEG_	N/A	22 h
IVIM‐NET_optim_	210 s	80 s
NATTEN‐17	8 d	20 min
SA‐17	21 h	2 h
SA‐1	20 h	20 min

*Note*: Since the conventional and Bayesian estimators are not trained, their training times are reported as N/A (not applicable). *s* is seconds, *h* is hours, *d* is days. To reduce computation time, LSQ and SEG were applied only to pixels within the brain, while the Bayesian approaches (BSP_brain_, BSP_tumor_, FBM_SEG_) were applied only to slices containing tumor, totaling 180 of the 392 in vivo slices.

## 4 Discussion

This study showed that spatially‐aware transformer architectures (NATTEN‐17, SA‐17) consistently produced significantly the most accurate parameter maps in simulations and, on visual inspection of the in vivo results, they clearly delineated tumor regions with smooth, detailed pseudo‐diffusion parameters. Among them, SA‐17 appeared visually to be the best at preserving anatomical boundaries. For tumor grading, SA‐17 achieved the best overall discrimination in whole‐tumor ROIs, although higher AUC values could be obtained by using tumor sub‐regions. These findings demonstrate the value of incorporating spatial context in parameter estimation to reduce noise and enhance clinical applicability in a clinical cohort. This underscores the importance of the choice of fitting method in estimating IVIM parameters for clinical usage, specifically in terms of tumor grading accuracy in gliomas.

### 4.1 Comparison of Fitting Methods

The superior performance of the transformer‐based methods likely arises from their ability to incorporate contextual information across large spatial extents. This apparently suppresses noise and mitigates the voxel‐wise parameter degeneracy inherent to the IVIM model [17]. SA‐17, which employs full self‐attention over patches, was particularly effective at preserving structural details, such as tissue heterogeneity. This suggests its potential for clinical decision‐making, such as guiding biopsies or optimizing radiotherapy treatment. Its larger receptive field also resulted in lower supervised parameter bias, whereas SA‐1, with its limited spatial context and effectively voxel‐wise network design, showed clear supervised bias toward the mean of the training distribution. In the original NATTEN‐17 implementation [17], NA was chosen for patches larger than 7 × 7 due to memory constraints when processing entire images during inference. However, by handling the image in smaller patch batches, SA‐17 can circumvent these memory limitations.

Although patch extraction increases the computational burden of SA‐17 compared to NATTEN‐17, its training is faster because substantially fewer attention blocks are required to achieve a comparable receptive field. NATTEN is formulated as a localized, translation‐equivariant attention operator with convolution‐like spatial constraints. Such locality limits direct modeling of long‐range dependencies and may impair aggregation of broader anatomical context. In MRI reconstruction, CNNs have been associated with attenuation of high‐frequency image components, reduced preservation of fine structural details, and blurred image details [35]. These findings support the interpretation that a strictly local, convolution‐like operator, such as in NATTEN‐17, may reduce preservation of edge‐like features when signal discrimination depends on broader spatial context. In contrast, SA‐17 considers all voxels in each self‐attention block equally, facilitating the recognition of important spatial context.

Importantly, the use of synthetic training data with uniformly sampled IVIM parameter distributions and patch‐based spatial training supports broader generalizability [17]. By avoiding training on anatomy‐ or tumor‐specific patient data, the networks are not implicitly tuned to the glioma cohort and can potentially be applied to other anatomical regions or clinical scenarios without retraining. Hence, our approach avoids a common limitation of supervised learning when applied to medical image analysis [36, 37].

NATTEN‐17 achieved marginally lower error metrics in simulations. Comparable trends were observed when training on Gaussian‐distributed data guided by literature values for the brain (see Supporting Information), suggesting that a uniform distribution, independent of prior knowledge, can serve as an adequate training set [17]. Note that although some estimators, including SA‐17, NATTEN‐17, and FBM, incorporate spatial information from neighboring voxels, this does not bias the comparison between methods because all estimators are provided with identical DWI signal data. Therefore, observed performance differences reflect the distinct processing strategies of each method rather than differences in the underlying signal or violations of voxel independence.

Among the Bayesian methods, the BSP_tumor_ approach was a good performer at discriminating tumor grade. The benefit of incorporating informative priors in IVIM fitting has been demonstrated in previous comparative studies, where choice of prior substantially influenced parameter robustness and accuracy, particularly under low SNR conditions [8, 15]. However, a major disadvantage of this BSP_tumor_ approach is that the extent of the tumor needs to be known prior to implementation. Conversely, the FBM framework, while producing visually smooth maps, showed significantly weaker discriminative performance for *f* and D* than the spatially‐aware networks. As spatial regularization reduces estimator variance, Bayesian smoothing approaches may attenuate genuine localized heterogeneity, thereby reducing sensitivity to pathological differences [8, 15], which does not ensure optimal clinical separability.

LSQ and SEG are by far the most widely‐used estimators in clinical IVIM studies due to their simplicity of implementation and broad generalizability [10, 38]. However, these estimators performed significantly worse than the spatially‐aware networks in several metrics. LSQ produced visually noisy maps, particularly for D*, reflecting its well‐known sensitivity to noise. SEG improved the estimation of *f* in some contexts, yet it still provided visually noisier maps than the advanced approaches, and significantly weaker discriminative power for D*.

IVIM‐NET_optim_ was the fastest approach, but showed apparent biases toward the mean of the imposed sigmoid constraints, especially for D* in regions of low SNR, as reported elsewhere [25]. These elevated D* values do not indicate overfitting, as IVIM‐NET_optim_ is trained directly on in vivo signals (the test data itself) using a self‐supervised strategy, so it cannot overfit the test data. SA‐1, in contrast, showed biases toward the mean of the training distribution. These biases may obscure variations of tissue heterogeneity, which is consistent with prior studies [4, 15, 16, 39]. The elevated D* observed for IVIM‐NET_optim_ and SA‐1 aligns with simulation results, particularly in white matter regions with low *f* and low SNR, where D* estimation is inherently uncertain. For SA‐1, this reflects the bias of voxel‐wise supervised learning under uncertainty. Expanding the receptive field, as in SA‐17, leverages spatial redundancy and substantially reduces this bias, producing more stable and physiologically plausible D* estimates.

Importantly, improved parameter fitting accuracy does not inherently guarantee superior diagnostic performance. In quantitative imaging biomarker research, technical metrics such as bias and precision are conceptually distinct from clinical task performance [40]. In the IVIM literature, methodological comparisons tend to focus on technical characteristics of the estimators, particularly bias and precision [4, 5, 8]. Few studies have explicitly examined the relationship between estimator choice and clinically relevant outcomes. Vidic et al. [9] compared Bayesian and conventional IVIM estimators in breast cancer, assessing not only parameter stability but also lesion discrimination. Similarly, Jalnefjord et al. [41] investigated how Bayesian and conventional IVIM estimators influenced discriminative ability in liver tumor applications. Building on this line of work, our study explicitly examines whether advanced modeling and DL approaches have measurable consequences for downstream diagnostic tasks. Our work therefore contributes new evidence to this ongoing discussion and helps clarify whether methodological improvements in IVIM fitting are clinically meaningful.

### 4.2 Clinical Outlook

These findings demonstrate that spatially‐aware IVIM estimators achieve better discriminative performance for tumor grading relative to conventional LSQ or SEG, particularly for perfusion‐related parameters. In this cohort, tumor grading captures relevant differences among subtypes, with glioblastomas classified as WHO Grade‐4 and mixed gliomas such as oligoastrocytomas typically classified as WHO Grade‐2 or Grade‐3 [29]. These grading distinctions may therefore be reflected in the performance of IVIM‐derived parameters.

Clinically, prior IVIM studies have almost universally relied on LSQ or SEG, with fewer reports using Bayesian approaches. In gliomas, for example, Hu et al. [42] employed SEG using sub‐region tumor ROIs and reported strong separation of low‐ (Grade 1 or Grade 2) and high‐grade (Grade 3 or Grade 4) tumors with AUC_D_ = 0.94, AUC_f_ = 0.90 and AUC_D*_ = 0.80. Shen et al. [43] found that in sub‐region glioma ROI analysis, the derived product *f* × D* yielded excellent grade separation (AUC ~0.98). Vidic et al. [9] found that Bayesian frameworks improved the discriminative power of pseudo‐diffusion parameters compared to conventional fitting in breast tumors (e.g., AUC_D_ = 0.91, AUC_f_ = 0.73 and AUC_D*_ = 0.64 for BSP_tumor_).

The spatially‐aware Grade‐4 vs. Grade‐2/3 discrimination results in the current study (AUC_D_ = 0.71, AUC_f_ = 0.65, AUC_D*_ = 0.78 for SA‐17) fall within the lower range of these previous studies, although with a whole‐tumor analysis strategy. Prior studies reporting higher AUC values frequently relied on subregion–based analyses or selected voxel subsets, which are known to enhance apparent grading performance. Consistent with this, we demonstrate in this work that adopting a subregion or percentile‐based analysis of IVIM parameters leads to improved grading accuracy, yielding results that are more directly comparable to the higher performance reported in the literature. These findings highlight that differences in reported performance are strongly influenced by both the estimation method and the tumor analysis strategy.

The percentile‐based analysis indicates that some non‐spatially‐aware methods show stronger discriminative performance for *f* at higher percentiles, reflecting sensitivity to extremal values within heterogeneous tumor regions. By contrast, the spatially‐aware transformer networks exhibit lower AUC for *f* at higher percentiles, which might reflect a trade‐off whereby spatial regularization and contextual integration de‐emphasize localized extreme perfusion values. Notably, the spatially‐aware networks retain strong discriminative performance for D* across most percentiles, suggesting that perfusion‐related differences between gradings may be more broadly discernible in D*, whereas *f* may reflect more localized heterogeneity that becomes apparent only at higher percentiles.

In this study, the spatially‐aware networks estimated significantly lower *f* values in Grade‐4 tumors compared to Grade‐2, contrary to expectations [27]. The *f* parameter has previously been proposed as a useful marker for distinguishing brain tumor grades [27]. MRI studies have demonstrated that cerebral blood volume correlates with the extent of neovascularization [44], and elevated perfusion is associated with higher tumor grade [45] and poorer prognosis [46]. High‐grade neoplasms typically develop a pathological microvascular network via neoangiogenesis to meet increased demands for nutrients and oxygen [47], suggesting that higher *f* may correspond with more aggressive tumors [41]. However, the results of the current study suggest the opposite trend for the spatially‐aware networks, potentially indicating that perfusion‐related information is more strongly captured by D*, which is elevated in Grade‐4 tumors. It is also possible that residual bias contributes to the high D* estimated by the spatially‐aware networks. The SEG results align with the expected pattern, showing higher *f* values for Grade‐4 tumors, also consistent with a previous study using a similar dataset [27]. Given the limited size of this study's cohort, these trends should be interpreted cautiously.

From an imaging perspective, the improved discriminative performance and markedly smoother IVIM parameter maps produced by the spatially‐aware estimators may facilitate more consistent visual interpretation, even in the absence of standardized grading criteria for radiologists. Reduced noise and improved spatial coherence can mitigate inter‐observer variability when IVIM maps are reviewed qualitatively, particularly for perfusion‐related parameters. In this sense, quantitative performance gains may translate into practical improvements in the interpretability of IVIM maps. We expect such improvements should positively impact formal tumor grading; however, this has not been explored explicitly in this study.

### 4.3 Limitations

The limited size, heterogeneity, and single‐center origin of the in vivo MRI data are key limitations of this study. The small size limited the statistical power, particularly for subgroup analyses by tumor grade, and may reduce the generalizability of the findings to broader populations or different acquisition protocols. Furthermore, the imbalance in grade distribution, with a predominance of Grade‐4 tumors, could skew grading performance metrics and obscure trends in lower‐grade gliomas. The ability of these algorithms to capture intratumoral heterogeneity, such as variations in *f*, should also be validated against histological ground truth, ideally using localized tumor biopsies stained for markers of neovascularization. Ideally such studies would include repeated acquisitions to permit further assessment of the robustness of the various IVIM parameter estimators.

To strengthen validation, multi‐center studies with larger, more balanced cohorts, encompassing tumors from different anatomical locations, are needed. Such studies would enable evaluation of the consistency of observed trends across scanners, institutions, acquisition settings, and tumor types. Such broader assessments would allow examination of reproducibility and potential site‐specific effects that cannot be resolved within a single cohort. The present findings may therefore provide feasibility insights that can inform the design and statistical planning of subsequent large‐scale investigations aimed at assessing generalizability.

An additional limitation of the DL–based estimators evaluated in this work is their dependence on a fixed diffusion acquisition protocol, as all networks were trained for a specific number and distribution of *b* values. Consequently, applying these networks to data acquired with different protocols would generally require retraining, which may limit immediate generalizability across sites. Recently proposed neural controlled differential equation (NCDE)–based approaches [48] enable sequence‐agnostic IVIM modeling, where a single network can accommodate varying *b*‐value samplings without retraining. While such approaches were not included in our comparative analysis, their formulation illustrates that architectural designs can be constructed to relax protocol‐specific constraints.

In addition, the field has recently seen substantial growth in the development of DL–based IVIM estimators, including other self‐supervised [20, 49, 50] and supervised [23, 51] networks. This rapidly expanding methodological landscape reflects strong interest in overcoming the instability and noise sensitivity of conventional IVIM fitting. However, it also means that a comprehensive comparison of all available DL approaches was beyond the scope of the present work. We therefore restricted our analysis to a representative subset of methods spanning conventional fitting, Bayesian regularization, voxel‐wise DL, and spatially‐aware transformer‐based estimators. While this selection captures major model‐fitting paradigms currently in use, it constitutes a limitation, as alternative network architectures, training strategies, or protocol‐agnostic designs may yield different performance characteristics.

## 5 Conclusion

By framing IVIM parameter estimation in terms of tumor grading accuracy, this study strengthened the case for spatially‐aware networks, particularly for extracting pseudo‐diffusion parameters. Leveraging spatial context allowed these networks to suppress noise while preserving fine structural details and tumor heterogeneity, yielding parameter maps that were both smoother and more anatomically coherent than those obtained with voxel‐wise methods. This balance between noise suppression and structural preservation improved the visual interpretability of IVIM maps, yielded significantly improved tumor grading accuracy, and may permit the integration of IVIM analysis into clinical decision‐making.

## Funding

Misha P. T. Kaandorp gratefully acknowledges support from the Swiss National Science Foundation (grant no. IZKSZ3_218590). Andras Jakab has received funding from the EMDO Foundation, Vontobel Foundation, Anna Müller‐Grocholski, and Prof. Max Cloetta Foundations. Peter T. While gratefully acknowledges support from the Research Council of Norway under FRIPRO Researcher Project 302624.

## Supporting information


**Figure S1:** Patch propagation for the spatially‐aware transformer networks. (A) NATTEN‐17: Comprises eight neighborhood‐attention (NA) blocks, each with a kernel size of 3 × 3. In each block, a convolution‐like attention mechanism propagates information from neighboring pixels. Each block in the sequence carries information from the input patch, and after the final block, only a single set (or pixel) is apparent that contains four nodes (1 × 4), representing the IVIM parameters (*S*_0_, *D*, *f*, D*). The propagation illustrated in red indicates the 3 × 3 attention window applied to the forward pixel; this same attention pattern is used for all pixels. The loss of NATTEN‐17 is computed between the predicted IVIM parameters of this final set and the simulated IVIM parameters used to generate the DWI signals for the center pixel of the input patch. (B) SA‐17: Contains 3 self‐attention (SA) blocks, where each pixel attends to all other pixels. Consequently, the last SA block still attends to all pixels in the patch, facilitating long‐range identification of similar signals. Self‐attention requires more memory due to the quadratic scaling of attention across all pixels. The propagation illustrated in green and blue indicates the 17 × 17 attention window applied to the forward pixel; this same attention pattern is used for all pixels. For SA‐17, the loss is computed between the predicted IVIM parameters of the center pixel in the output patch (1 × 4) and the simulated parameters used to generate the DWI signals for the center pixel of the input patch. During inference, individual batches of patches for each voxel are fed to the network to manage memory usage.
**Figure S2:** Example fractal‐noise‐based IVIM parameter maps, root mean square error (RMSE) maps (calculated between the estimated DWI signal and the ground truth DWI signal), and absolute error maps, estimated for four (FBM_LSQ_, IVIM‐NET_orig_, SA‐17 rg, SA‐1 rg) of the evaluated IVIM fitting methods. “rg” stands for random‐gaussian. (D*, pseudo‐diffusion coefficient; *D*, diffusion coefficient; *f*, perfusion fraction).
**Figure S3:** Boxplots showing the median absolute percentage error (MDAPE) and median percentage error (MDPE) for each IVIM parameter for four (FBM_LSQ_, IVIM‐NET_orig_, SA‐17 rg, SA‐1 rg) of the evaluated IVIM fitting methods, calculated on the entire IVIM fractal‐based test set (40 sets of fractal‐noise IVIM parameter maps). “rg” stands for random‐gaussian. To provide insight into network stability, the error metrics were computed for the spatially‐aware transformer networks at each epoch of the last 20 epochs of the entire training. For IVIM‐NET, these were run 20 times. Gray represents conventional and Bayesian estimators, blue represents voxel‐wise DL‐based estimators, orange represents spatially‐aware DL estimators. A Mann–Whitney *U* test with Bonferroni correction was used to test for significant differences between groups (indicated by bars above the plots). Values printed above the boxplots indicate median values that are outside the displayed plot range. (D*, pseudo‐diffusion coefficient; *D*, diffusion coefficient; *f*, perfusion fraction).
**Figure S4:** IVIM parameter maps and root mean square error (RMSE) maps (calculated between the estimated DWI signal and the ground truth DWI signal) for a representative slice of a Grade‐2 oligoastrocytoma patient. The maps are estimated for four (FBM_LSQ_, IVIM‐NET_orig_, SA‐17 rg, SA‐1 rg) of the evaluated IVIM fitting methods. “rg” stands for random‐gaussian. Also shown is the *b* = 0 image (*top left*), where the purple arrow indicates the location of the oligoastrocytoma. (D*, pseudo‐diffusion coefficient; *D*, diffusion coefficient; *f*, perfusion fraction).
**Figure S5:** IVIM parameter maps and root mean square error (RMSE) maps (calculated between the estimated DWI signal and the ground truth DWI signal) for a representative slice of a Grade‐4 glioblastoma patient. The maps are estimated for four (FBM_LSQ_, IVIM‐NET_orig_, SA‐17 rg, SA‐1 rg) of the evaluated IVIM fitting methods. “rg” stands for random‐gaussian. Also shown is the *b* = 0 image (*top left*), where the purple arrow indicates the location of the glioblastoma. Note the strong edge preservation for the SA‐17 random‐gaussian network, particularly for *f* and the high D*, comparable to SA‐17 trained on a uniform distribution in Figure 4 of the main manuscript. (D*, pseudo‐diffusion coefficient; *D*, diffusion coefficient; *f*, perfusion fraction).
**Figure S6:** Box‐and‐whisker plots for the IVIM parameters of all pooled estimates within the whole tumor ROIs for four (FBM_LSQ_, IVIM‐NET_orig_, SA‐17 rg, SA‐1 rg) of the evaluated IVIM fitting methods, across the different tumor grades and contralateral control region. “rg” stands for random‐gaussian. A Mann–Whitney *U* test with Bonferroni correction was used to test for significant differences between groups (indicated by bars above the plots). (D*, pseudo‐diffusion coefficient; *D*, diffusion coefficient; *f*, perfusion fraction).
**Figure S7:** ROC curves and AUC values for differentiating Grade‐4 from combined Grade‐2 and Grade‐3 tumors for four (FBM_LSQ_, IVIM‐NET_orig_, SA‐17 rg, SA‐1 rg) of the evaluated IVIM fitting methods using (A) parameter estimates from all voxels within the whole tumor ROIs, and (B) percentile values (5%, 25%, 50%, 75%, 95%) from the estimates for each tumor ROI. “rg” stands for random‐gaussian. The AUC values in (B) suggest improved grading accuracy may be possible by focusing on subregions with low *D*, high *f*, and/or high D*. (D*, pseudo‐diffusion coefficient; *D*, diffusion coefficient; *f*, perfusion fraction).
**Figure S8:** Comparison of diagnostic performance against simulation bias for four (FBM_LSQ_, IVIM‐NET_orig_, SA‐17 rg, SA‐1 rg) of the evaluated IVIM fitting methods. “rg” stands for random‐gaussian. For each IVIM parameter, the receiver operating characteristic area under the curve (ROC‐AUC) for differentiating Grade‐4 from combined Grade‐2 and Grade‐3 tumors is plotted against the median percentage error (MDPE) obtained from the fractal‐based simulation test set. Each marker represents one fitting method. Gray represents conventional and Bayesian estimators, blue represents voxel‐wise DL‐based estimators, orange represents spatially‐aware DL estimators. The horizontal dashed line indicates zero bias (MDPE = 0). The D* panel does not include IVIM‐NET_orig_ because of its extremely high bias (128%). (D*, pseudo‐diffusion coefficient; *D*, diffusion coefficient; *f*, perfusion fraction).
**Table S1:** Computation times for four (FBM_LSQ_, IVIM‐NET_orig_, SA‐17 rg, SA‐1 rg) of the evaluated IVIM fitting methods when applied to the cohort of 20 glioma patients. Since the Bayesian estimators are not trained, their training times are reported as N/A (not applicable). *s* is seconds, *h* is hours, *d* is days. To reduce computation time, the Bayesian approaches (FBM_LSQ_) were applied only to slices containing tumor, totaling 180 of the 392 in vivo slices.

## Data Availability

The training code for the SA‐17 and NATTEN‐17 networks, for training on either the “random‐uniform” or “random‐gaussian” patches, is available on GitHub: https://github.com/Mishakaandorp/Incorporating_spatial_information_in_deep_learning_parameter_estimation.
