# Supplementary material for: A Comparative Study of IVIM‐MRI Fitting Techniques in Glioma Grading: Conventional, Bayesian, and Voxel‐Wise and Spatially‐Aware Deep Learning Approaches
Source: J Magn Reson Imaging. 2026 Mar 30;64(1):184–200. doi: 10.1002/jmri.70301 (PMC13254000; doi:10.1002/jmri.70301)
Supplement: Supplementary file 1 — Figure S1: Patch propagation for the spatially‐aware transformer networks. (A) NATTEN‐17: Comprises eight neighborhood‐attention (NA) blocks, each with a kernel size of 3 × 3. In each block, a convolution‐like attention mechanism propagates information from neighboring pixels. Each block in the sequence carries information from the input patch, and after the final block, only a single set (or pixel) is apparent that contains four nodes (1 × 4), representing the IVIM parameters (S 0, D, f, D*). The propagation illustrated in red indicates the 3 × 3 attention window applied to the forward pixel; this same attention pattern is used for all pixels. The loss of NATTEN‐17 is computed between the predicted IVIM parameters of this final set and the simulated IVIM parameters used to generate the DWI signals for the center pixel of the input patch. (B) SA‐17: Contains 3 self‐attention (SA) blocks, where each pixel attends to all other pixels. Consequently, the last SA block still attends to all pixels in the patch, facilitating long‐range identification of similar signals. Self‐attention requires more memory due to the quadratic scaling of attention across all pixels. The propagation illustrated in green and blue indicates the 17 × 17 attention window applied to the forward pixel; this same attention pattern is used for all pixels. For SA‐17, the loss is computed between the predicted IVIM parameters of the center pixel in the output patch (1 × 4) and the simulated parameters used to generate the DWI signals for the center pixel of the input patch. During inference, individual batches of patches for each voxel are fed to the network to manage memory usage. Figure S2: Example fractal‐noise‐based IVIM parameter maps, root mean square error (RMSE) maps (calculated between the estimated DWI signal and the ground truth DWI signal), and absolute error maps, estimated for four (FBMLSQ, IVIM‐NETorig, SA‐17 rg, SA‐1 rg) of the evaluated IVIM fitting methods. “rg” stands for random‐gau [file JMRI-64-184-s001.docx]

**S1. Additional Methodology**

**Datasets**

**Synthetic Data**

Here we describe the generation of the fractal-noise-based IVIM parameter maps as proposed by Kaandorp et al. [1].

Fractal noise, which should not be confused with Rician noise inherent to MRI signals, is formed by combining several layers (or octaves) of Perlin noise [2]. Perlin noise is a continuous, wave-like algorithm that produces gradient noise and can generate many random patterns with a distribution similar to a Gaussian curve. The process of generating Perlin noise typically involves three main steps: (1) creating an n-dimensional grid of random gradient vectors, (2) calculating the dot product between these gradients and their offsets, and (3) interpolating the resulting values.

First, one fractal-based map was used for creating masks corresponding to three primary brain tissue classes: gray matter (GM), white matter (WM), and cerebrospinal fluid (CSF). The fractal map was normalized to a range of 0 to 1, after which specific intensity intervals were assigned to each tissue type: (0<CSF<0.2; 0.2<GM<0.5; 0.5<WM<0.8; 0.8<CSF<1).

For each tissue class and each IVIM parameter (S₀, D, f, D*), separate fractal-based maps were generated, resulting in a total of 12 parameter-specific maps. Here, the S_0_ is the signal at *b* = 0. These maps were subsequently scaled according to literature-informed parameter ranges and combined with the tissue masks to create composite maps for each parameter. CSF parameter ranges were set between 0.5≤S_0_≤1, 1×10^-3^≤D≤3×10^-3^ mm^2^/s, 25≤f≤50%, and 20×10^-3^≤D*≤50×10^-3^ mm^2^/s; WM parameters between 0.05≤S_0_≤0.25, 0.3×10^-3^≤D≤1.3×10^-3^ mm^2^/s, 1≤f≤10%, and 3×10^-3^≤D*≤15×10^-3^ mm^2^/s; and GM parameters between 0.2≤S_0_≤0.5, 0.5×10^-3^≤D≤2×10^-3^ mm^2^/s, 5≤f≤25%, and 3×10^-3^≤D*≤30×10^-3^ mm^2^/s. Simulated diffusion-weighted imaging (DWI) signals were subsequently generated on a per-pixel basis using the corresponding parameter values in Eq. (1) across the 16 *b* values used for the in vivo data (see Section 2.1.2 of the main manuscript). Rician noise was added to the signals, such that S_0_=1 corresponded to an SNR of 200. The final synthetic test dataset comprised 40 unique parameter map sets, each with dimensions of 128×128 voxels.

**Fitting Methods**

Below we describe the methods for three of the estimators: IVIM-NET_orig_, as well as SA-17 and SA-1 trained on the ‘random-gaussian’ synthetic data.

***IVIM-NET_orig_***

This is the first self-supervised deep learning model for IVIM parameter estimation, proposed by Barbieri et al. [3], which employs a multi-layer perceptron (MLP) with three hidden layers, each containing the same number of hidden units (#units, neurons) as the number of *b* values. Exponential linear unit (ELU) activations are used in the hidden layers, and absolute value functions constrain the output to non-negative values, mitigating instability from negative predictions. The network outputs three parameters (D, f, D*).

We trained IVIM-NET_orig_ with 500 batches per epoch and batch size 128 using an Adam optimizer with a learning rate of 10^-2^. Early stopping was applied if the validation loss failed to improve for 10 consecutive epochs, as per the original work. Further details can be found in Barbieri et al. [3] and Kaandorp et al. [4].

***SA-17 random-gaussian (rg)***

This is the same network architecture as SA-17 but trained on 17×17 random patches with parameter values drawn from Gaussian (random-gaussian) rather than uniform distributions. Each patch contained a random number of spatially correlated neighbors around the center voxel. However, the tissue type corresponding to the center pixel and its correlated neighbors was assigned at random (i.e. GM, WM or CSF). If a particular tissue type (e.g., WM) was chosen for the center voxel and its correlated neighbors, the remaining uncorrelated neighbors were randomly assigned to one of the other two tissue classes (e.g. GM or CSF), ensuring that each patch contained at most two tissue types.

The key distinction from the random-uniform setup lies in the parameter sampling. Instead of drawing parameter values uniformly across the defined ranges, random-gaussian sampling used truncated Gaussian distributions designed to approximate the parameter distributions of the IVIM fractal-based test set, which were guided by literature, as per Kaandorp et al. [1]. For each tissue type and parameter, the Gaussian mean was set to the midpoint of its predefined range, and the standard deviation to half that range. Truncation ensured the sampled values remained within the same bounds as the uniform case. For example, for D* in WM, the Gaussian distribution had a mean 9×10^-3^ mm^2^/s, a standard deviation of 6×10^-3^ mm^2^/s, and was truncated to 3×10^-3^≤D*≤100×10^-3^ mm^2^/s. IVIM signals were then simulated from these parameter values with added Rician noise, as described for ‘random-uniform’ in Section 2.2.3 of the main manuscript.

***SA-1 random-gaussian***

This is an identical network to SA-17 random-gaussian (i.e. 3 SA blocks), except that it is applied to 1x1 random-gaussian patches (or single pixels), effectively reducing the architecture to a supervised voxel-wise model (i.e. with no access to spatial information), but with attention blocks.

**Methodological figure on NATTEN-17 and SA-17**

**
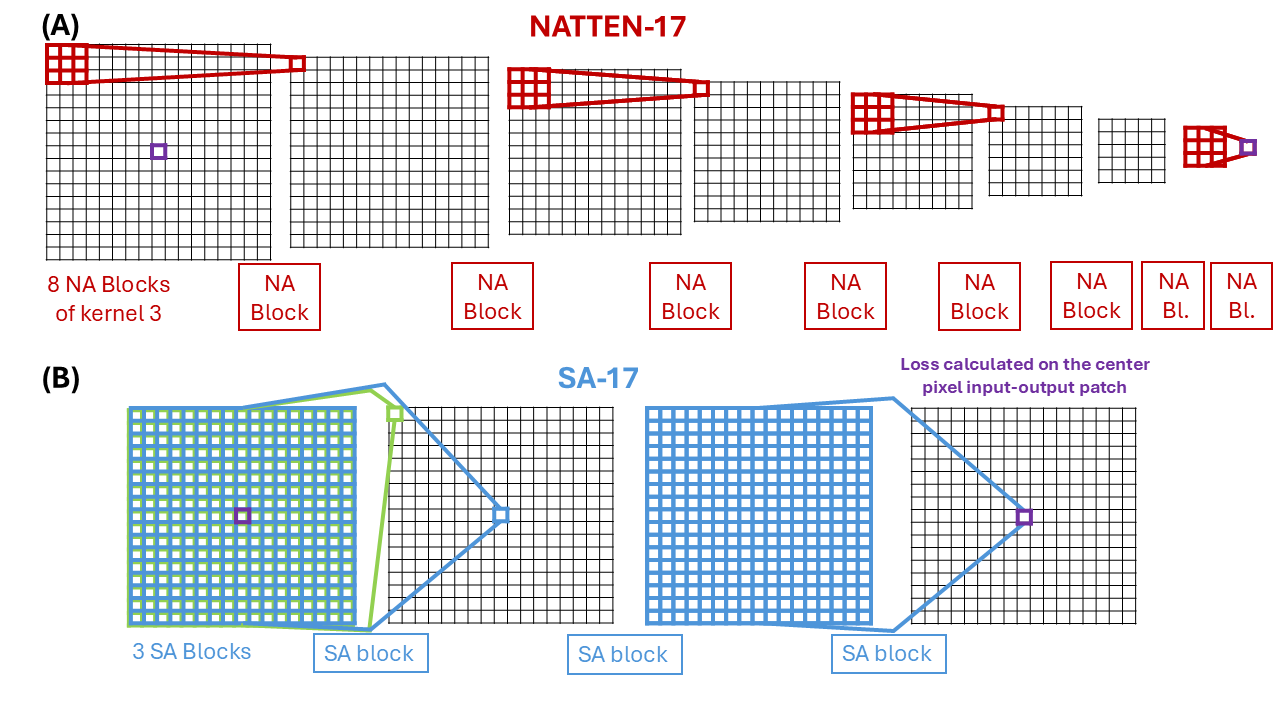
**

**Figure S1**: Patch propagation for the spatially-aware transformer networks. **(A)** NATTEN-17: Comprises 8 neighborhood-attention (NA) blocks, each with a kernel size of 3×3. In each block, a convolution-like attention mechanism propagates information from neighboring pixels. Each block in the sequence carries information from the input patch, and after the final block, only a single set (or pixel) is apparent that contains four nodes (1x4), representing the IVIM parameters (S0, D, f, D*). The propagation illustrated in red indicates the 3×3 attention window applied to the forward pixel; this same attention pattern is used for all pixels. The loss of NATTEN-17 is computed between the predicted IVIM parameters of this final set and the simulated IVIM parameters used to generate the DWI signals for the center pixel of the input patch. **(B)** SA-17: Contains 3 self-attention (SA) blocks, where each pixel attends to all other pixels. Consequently, the last SA block still attends to all pixels in the patch, facilitating long-range identification of similar signals. Self-attention requires more memory due to the quadratic scaling of attention across all pixels. The propagation illustrated in green and blue indicates the 17×17 attention window applied to the forward pixel; this same attention pattern is used for all pixels. For SA-17, the loss is computed between the predicted IVIM parameters of the center pixel in the output patch (1x4) and the simulated parameters used to generate the DWI signals for the center pixel of the input patch. During inference, individual batches of patches for each voxel are fed to the network to manage memory usage.

**S2. Additional Results**

Below are the results for the estimators: FBM_LSQ_ (described in main manuscript), IVIM-NET_orig_, SA-17 random-gaussian, and SA-1 random-gaussian.

**Evaluation in Synthetic Data**

**Table S1:** Computation times for four (FBM_LSQ_, IVIM-NET_orig_, SA-17 rg, SA-1 rg) of the evaluated IVIM fitting methods when applied to the cohort of 20 glioma patients. Since the Bayesian estimators are not trained, their training times are reported as N/A (not applicable). *s* is seconds, *h* is hours, *d* is days. To reduce computation time, the Bayesian approaches (FBM_LSQ_) were applied only to slices containing tumor, totaling 180 of the 392 in vivo slices.

|  | **Training** | **Inference/application** |
| --- | --- | --- |
| **FBM_LSQ_** | N/A | 81h |
| **IVIM-NET_orig_** | 100s | 80s |
| **SA-17 rg** | 24h | 2h |
| **SA-1 rg** | 19h | 20min |


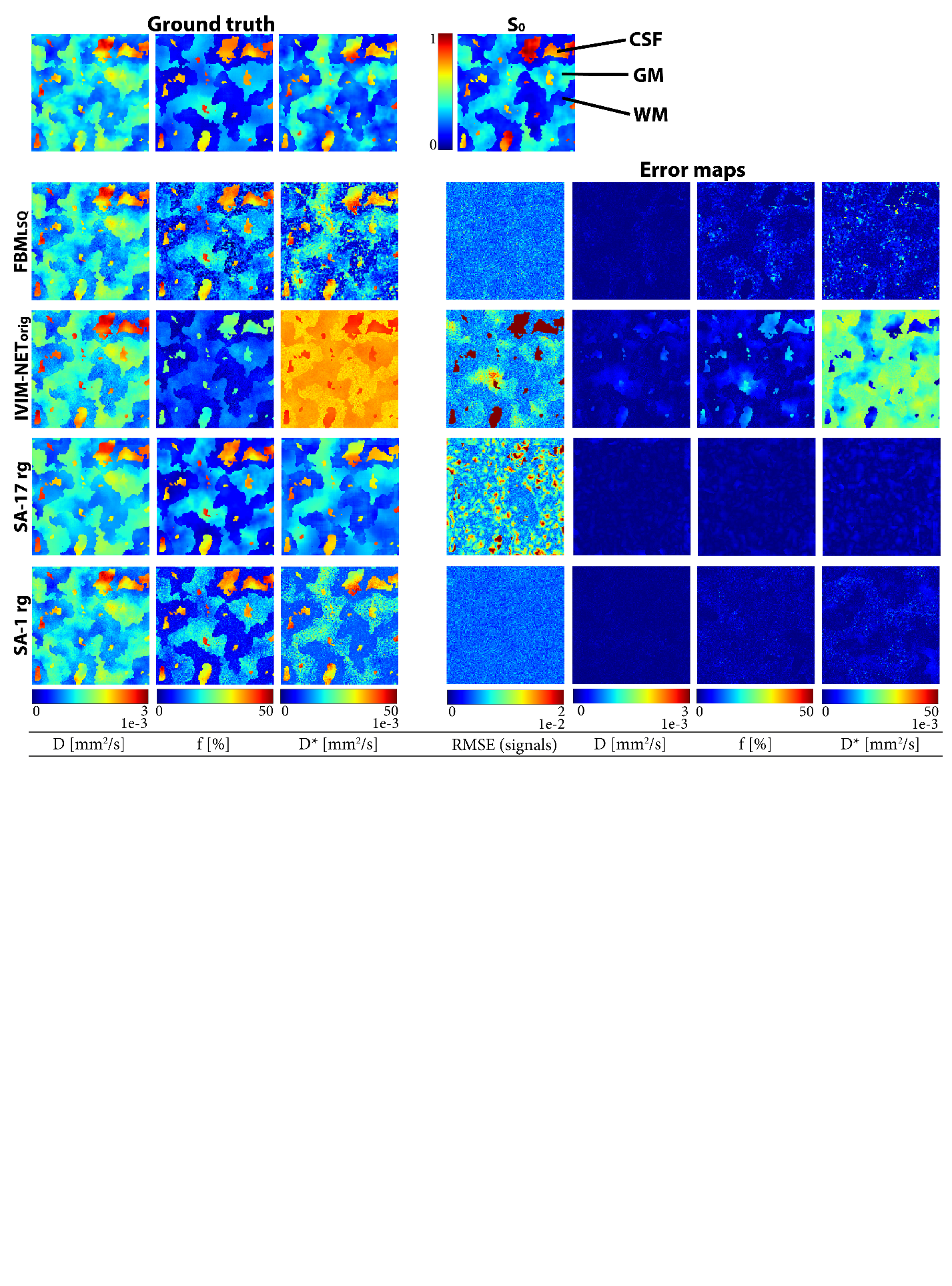


**Figure S2**: Example fractal-noise-based IVIM parameter maps, root mean square error (RMSE) maps (calculated between the estimated DWI signal and the ground truth DWI signal), and absolute error maps, estimated for four (FBM_LSQ_, IVIM-NET_orig_, SA-17 rg, SA-1 rg) of the evaluated IVIM fitting methods. ‘rg’ stands for random-gaussian. (D*, pseudo-diffusion coefficient; D, diffusion coefficient; f, perfusion fraction).


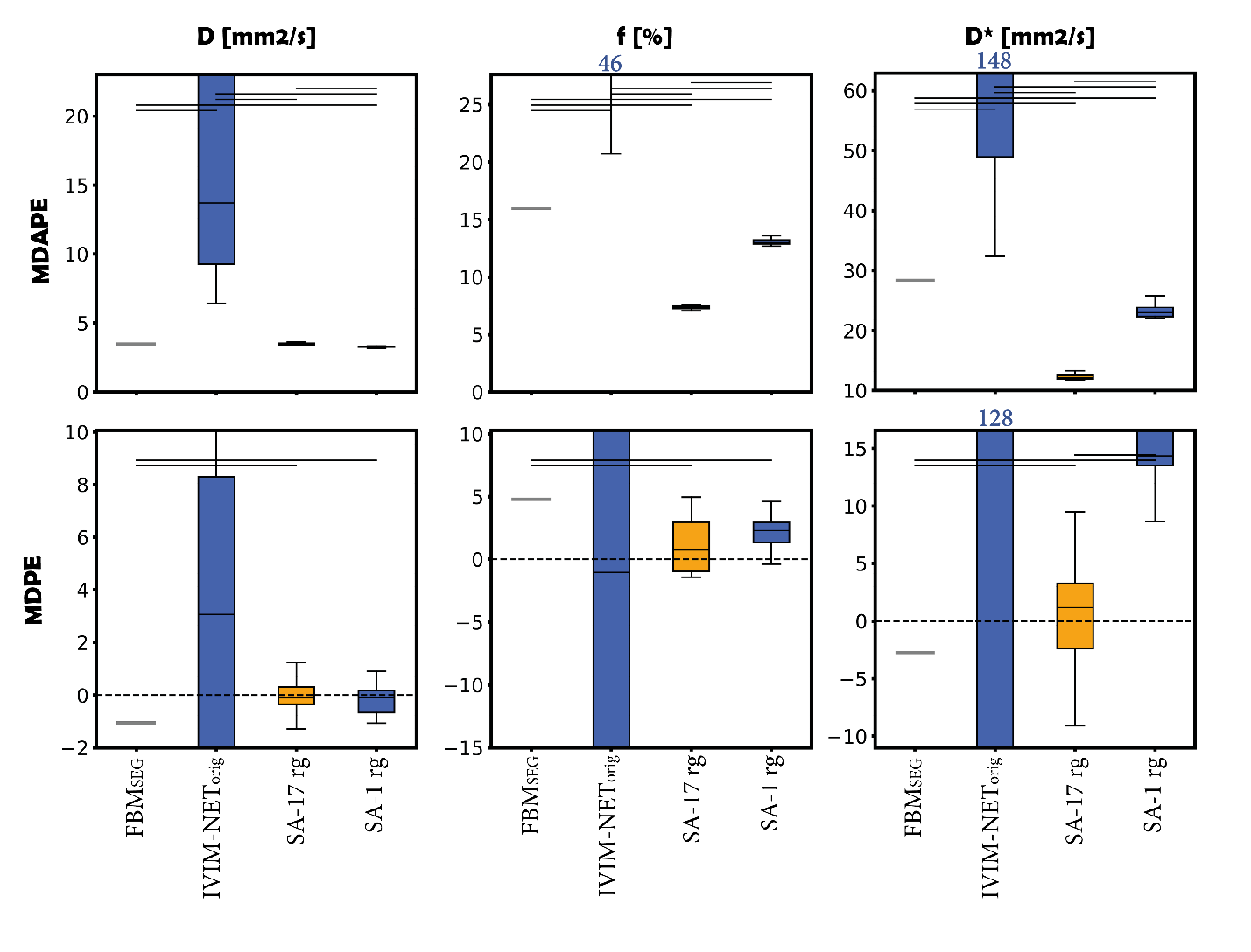


**Figure S3**: Boxplots showing the median absolute percentage error (MDAPE) and median percentage error (MDPE) for each IVIM parameter for four (FBM_LSQ_, IVIM-NET_orig_, SA-17 rg, SA-1 rg) of the evaluated IVIM fitting methods, calculated on the entire IVIM fractal-based test set (40 sets of fractal-noise IVIM parameter maps). ‘rg’ stands for random-gaussian. To provide insight into network stability, the error metrics were computed for the spatially-aware transformer networks at each epoch of the last 20 epochs of the entire training. For IVIM-NET, these were run 20 times. Gray represents conventional and Bayesian estimators, blue represents voxel-wise DL-based estimators, orange represents spatially-aware DL estimators. A Mann-Whitney U test with Bonferroni correction was used to test for significant differences between groups (indicated by bars above the plots). Values printed above the boxplots indicate median values that are outside the displayed plot range. (D*, pseudo-diffusion coefficient; D, diffusion coefficient; f, perfusion fraction).

**Tumor Cohort Analysis**

**
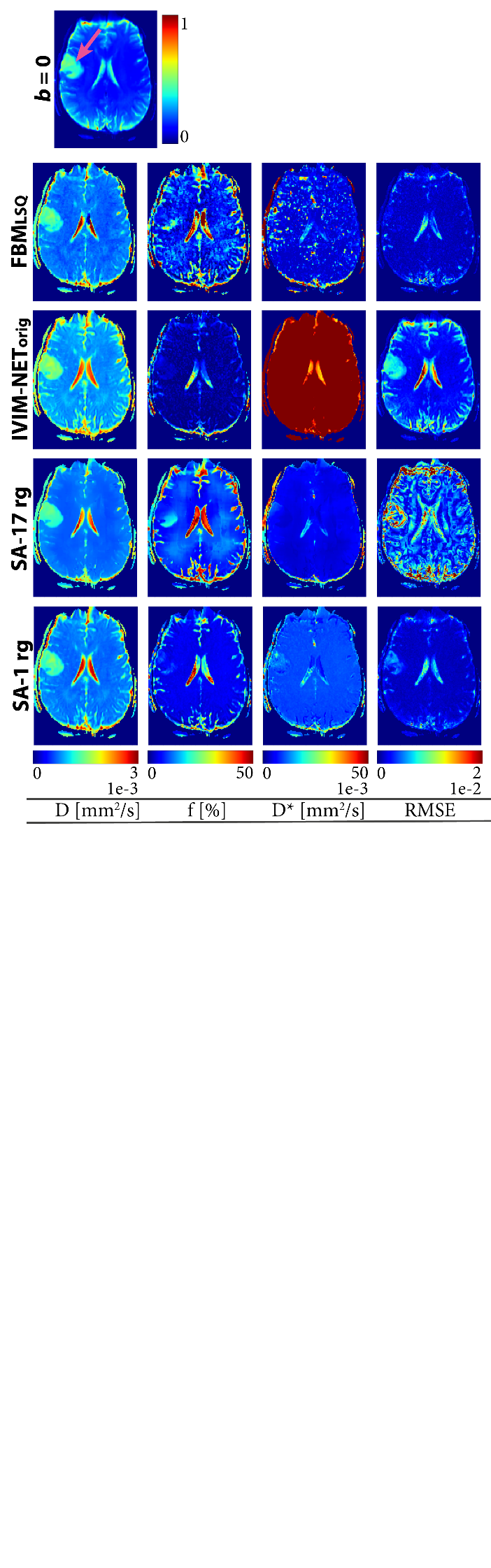
**

**Figure S4**: IVIM parameter maps and root mean square error (RMSE) maps (calculated between the estimated DWI signal and the ground truth DWI signal) for a representative slice of a Grade-2 oligoastrocytoma patient. The maps are estimated for four (FBM_LSQ_, IVIM-NET_orig_, SA-17 rg, SA-1 rg) of the evaluated IVIM fitting methods. ‘rg’ stands for random-gaussian. Also shown is the *b* = 0 image (*top left*), where the purple arrow indicates the location of the oligoastrocytoma. (D*, pseudo-diffusion coefficient; D, diffusion coefficient; f, perfusion fraction).

**
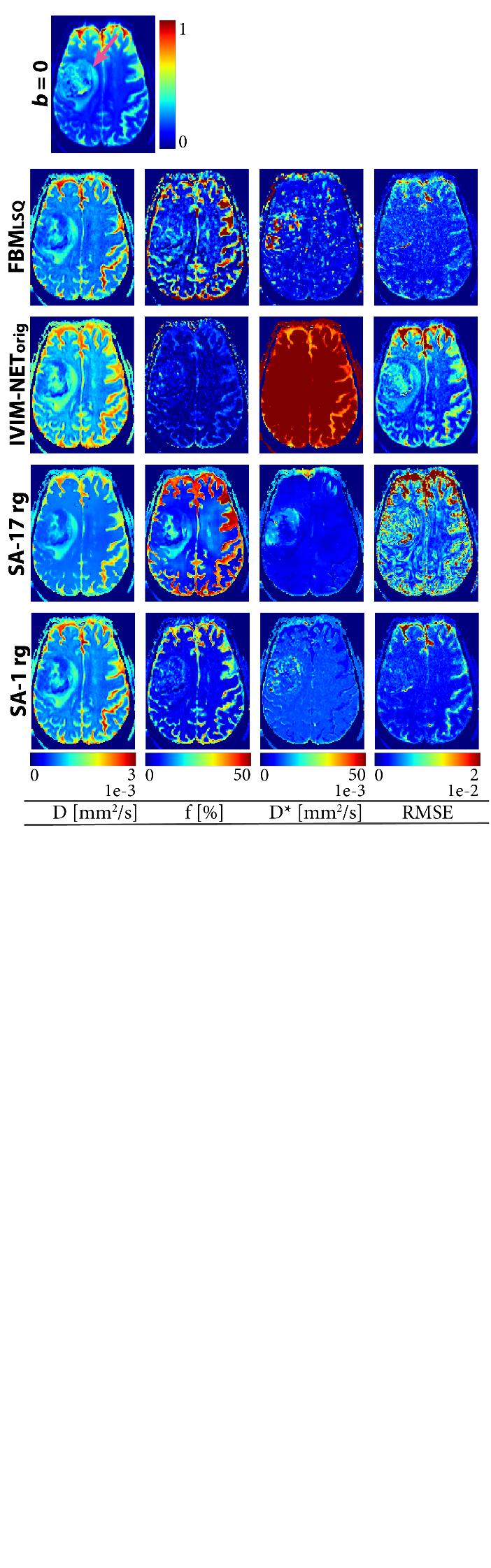
**

**Figure S5**: IVIM parameter maps and root mean square error (RMSE) maps (calculated between the estimated DWI signal and the ground truth DWI signal) for a representative slice of a Grade-4 glioblastoma patient. The maps are estimated for four (FBM_LSQ_, IVIM-NET_orig_, SA-17 rg, SA-1 rg) of the evaluated IVIM fitting methods. ‘rg’ stands for random-gaussian. Also shown is the *b* = 0 image (*top left*), where the purple arrow indicates the location of the glioblastoma. Note the strong edge preservation for the SA-17 random-gaussian network, particularly for f and the high D*, comparable to SA-17 trained on a uniform distribution in Fig. 4 of the main manuscript. (D*, pseudo-diffusion coefficient; D, diffusion coefficient; f, perfusion fraction).


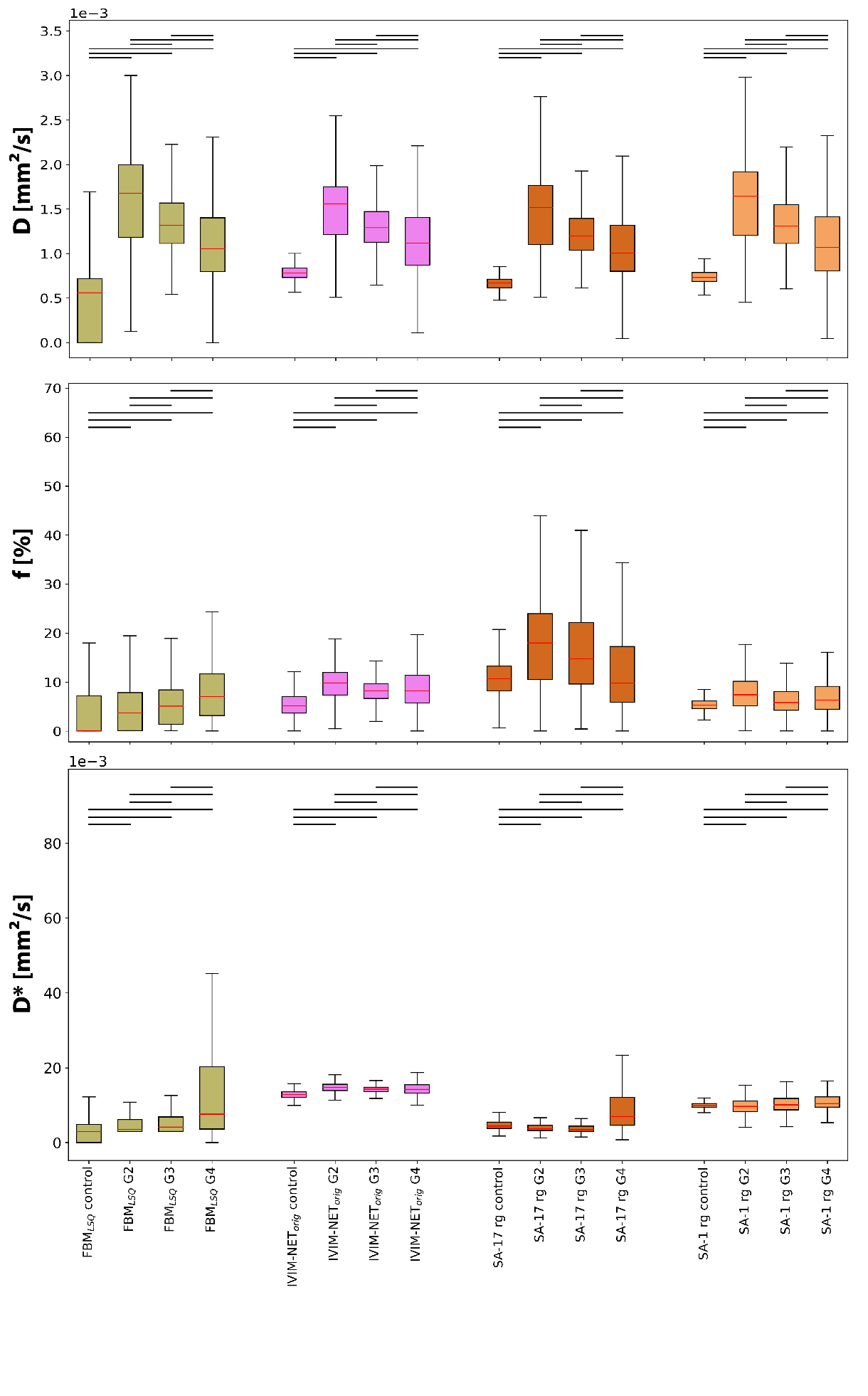


**Figure S6**: Box-and-whisker plots for the IVIM parameters of all pooled estimates within the whole tumor ROIs for four (FBM_LSQ_, IVIM-NET_orig_, SA-17 rg, SA-1 rg) of the evaluated IVIM fitting methods, across the different tumor grades and contralateral control region. ‘rg’ stands for random-gaussian. A Mann-Whitney U test with Bonferroni correction was used to test for significant differences between groups (indicated by bars above the plots). (D*, pseudo-diffusion coefficient; D, diffusion coefficient; f, perfusion fraction).


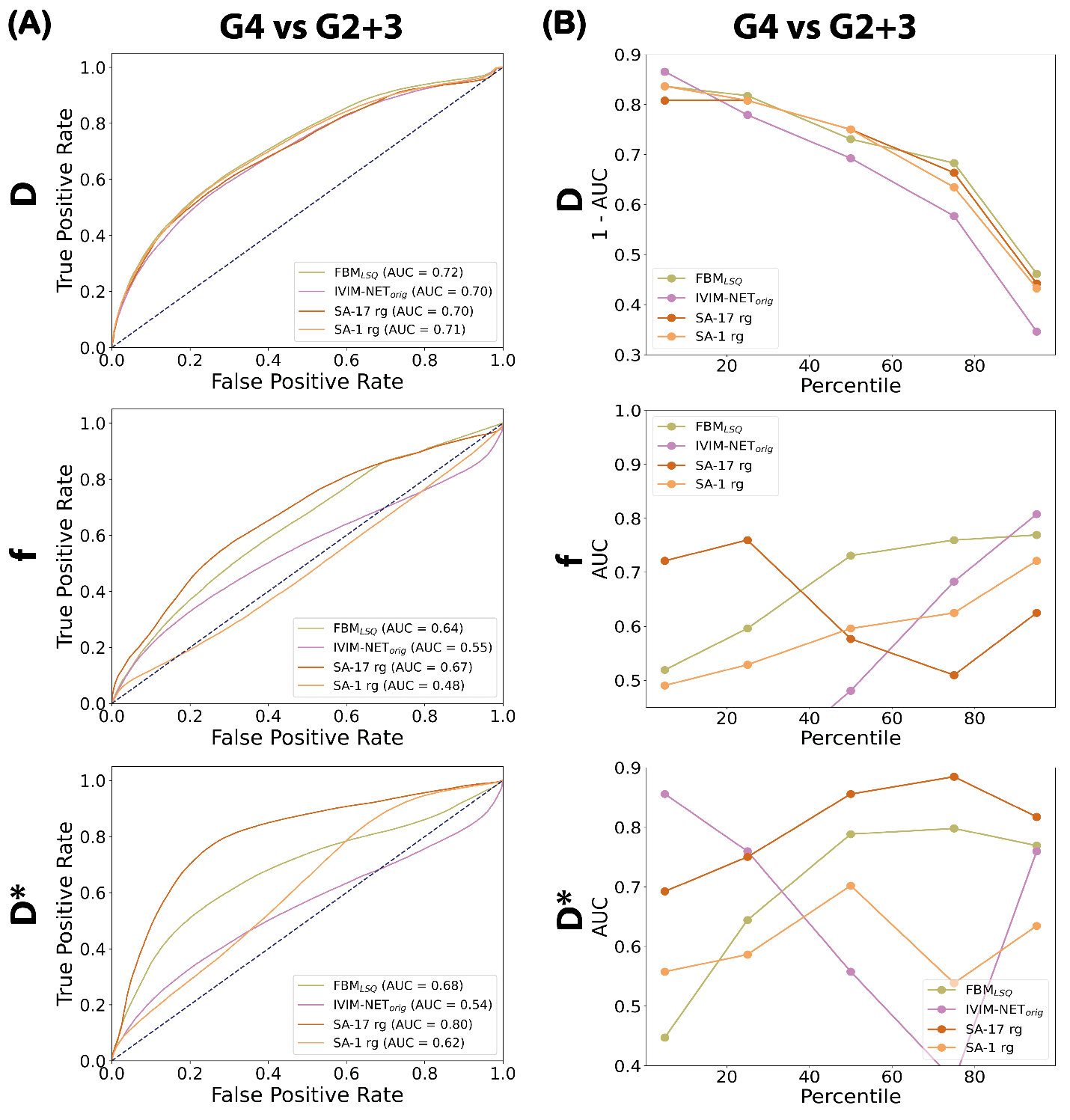


**Figure S7**: ROC curves and AUC values for differentiating Grade-4 from combined Grade-2 and Grade-3 tumors for four (FBM_LSQ_, IVIM-NET_orig_, SA-17 rg, SA-1 rg) of the evaluated IVIM fitting methods using (A) parameter estimates from all voxels within the whole tumor ROIs, and (B) percentile values (5%, 25%, 50%, 75%, 95%) from the estimates for each tumor ROI. ‘rg’ stands for random-gaussian. The AUC values in (B) suggest improved grading accuracy may be possible by focusing on subregions with low D, high f, and/or high D*. (D*, pseudo-diffusion coefficient; D, diffusion coefficient; f, perfusion fraction).

**
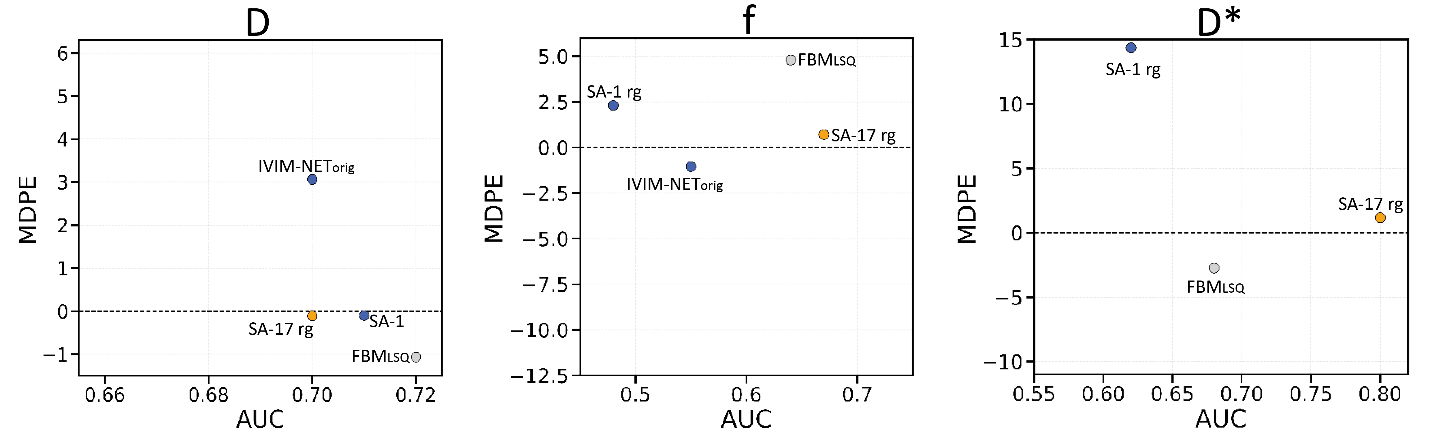
**

**Figure S8**: Comparison of diagnostic performance against simulation bias for four (FBM_LSQ_, IVIM-NET_orig_, SA-17 rg, SA-1 rg) of the evaluated IVIM fitting methods. ‘rg’ stands for random-gaussian. For each IVIM parameter, the receiver operating characteristic area under the curve (ROC-AUC) for differentiating Grade-4 from combined Grade-2 and Grade-3 tumors is plotted against the median percentage error (MDPE) obtained from the fractal-based simulation test set. Each marker represents one fitting method. Gray represents conventional and Bayesian estimators, blue represents voxel-wise DL-based estimators, orange represents spatially-aware DL estimators. The horizontal dashed line indicates zero bias (MDPE = 0). The D* panel does not include IVIM-NET_orig_ because of its extremely high bias (128%). (D*, pseudo-diffusion coefficient; D, diffusion coefficient; f, perfusion fraction).

**References**

[1] M.P.T. Kaandorp, F. Zijlstra, D. Karimi, A. Gholipour, P.T. While, Incorporating spatial information in deep learning parameter estimation with application to the intravoxel incoherent motion model in diffusion-weighted MRI, Med. Image Anal. 101 (2025). https://doi.org/10.1016/j.media.2024.103414.

[2] K. Perlin, Image Synthesizer., Computer Graphics (ACM) 19 (1985) 287–296. https://doi.org/10.1145/325165.325247.

[3] S. Barbieri, O.J. Gurney-Champion, R. Klaassen, H.C. Thoeny, Deep learning how to fit an intravoxel incoherent motion model to diffusion-weighted MRI, Magn. Reson. Med. 83 (2020) 312–321. https://doi.org/10.1002/mrm.27910.

[4] M.P.T. Kaandorp, S. Barbieri, R. Klaassen, H.W.M. van Laarhoven, H. Crezee, P.T. While, A.J. Nederveen, O.J. Gurney-Champion, Improved unsupervised physics-informed deep learning for intravoxel incoherent motion modeling and evaluation in pancreatic cancer patients., Magn. Reson. Med. 86 (2021) 2250–2265. https://doi.org/https://dx.doi.org/10.1002/mrm.28852.
